# Plant Tissue Structure and Degree of Milling as Determinants of Starch Digestibility and Postprandial Metabolic Responses to Cereal and Legume Products

**DOI:** 10.3390/nu18193175

**Published:** 2026-09-26

**Authors:** Maria-Christina Kanata, Stavroula Koroyannaki, Vaios T. Karathanos, Amalia E. Yanni

**Affiliations:** Department of Nutrition and Dietetics, Harokopio University, 70 El. Venizelou Ave., 17676 Athens, Greece; mckanata@hua.gr (M.-C.K.); ds218025@hua.gr (S.K.); ayanni@hua.gr (A.E.Y.)

**Keywords:** food matrix, degree of milling, particle size, cellular integrity, starch digestibility, resistant starch, postprandial glycemia, incretin hormones

## Abstract

**Background**: Growing evidence suggests that the structural integrity of plant tissues is an important determinant of starch digestibility and postprandial metabolic responses in cereals, legumes, and starch-rich foods. This narrative review evaluates the influence of botanical tissue architecture and the degree of milling on physicochemical properties, product quality, starch digestibility, and metabolic responses in cereal- and legume-based foods. **Methods**: The evidence was critically synthesized from studies on physicochemical characterization, in vitro starch digestibility, acute postprandial and long-term human intervention studies, and relevant systematic reviews and meta-analyses. **Results**: The preservation of intact cells, cellular aggregates, and coarser flour fractions limits water penetration and delays starch swelling and gelatinization, whereas extensive milling increases starch damage and susceptibility to enzymatic hydrolysis. Botanical origin further influences these effects, with legumes generally exhibiting lower starch digestibility than cereals because of their thicker and less permeable cell walls. Increasing the proportion of coarse flour fractions may also impair loaf volume, texture, processing performance, and consumer acceptability. Acute postprandial human intervention studies typically report attenuated glycemic responses following consumption of structurally preserved cereal- and legume-based foods, with several studies also showing improved insulinemic and incretin responses. However, human evidence is more heterogeneous than the relatively consistent in vitro evidence for slower starch hydrolysis with greater preservation of cellular structure, and these effects do not necessarily translate into lower postprandial glycemic responses. Evidence regarding appetite regulation and long-term metabolic outcomes remains limited or inconsistent. **Conclusions**: Cellular integrity and botanical structure are important but under-recognized determinants of carbohydrate functionality. Preserving plant tissue structure by controlling the degree of milling may contribute to the modulation of starch digestibility and postprandial metabolic responses. Integrating structural characteristics alongside nutrient composition may support the development of healthier cereal- and legume-based foods while maintaining technological functionality, consumer acceptability, and metabolic health.

## 1. Introduction

Increasing evidence indicates that the physiological effects of foods are determined not only by their nutrient composition but also by their structural organization and matrix properties. The physical arrangement of nutrients within the food matrix plays a central role in regulating digestibility, bioaccessibility, and bioavailability, thereby influencing postprandial metabolic responses. Conventional nutritional approaches typically classified foods according to chemical composition, with less emphasis on the structural features governing nutrient release during digestion and subsequent absorption. Consequently, foods with similar macronutrient compositions may elicit markedly different metabolic responses due to differences in their structural, physicochemical, and sensory properties across multiple length scales. In edible plants, this hierarchy extends from intact tissues to cellular and subcellular structures, including cell walls (10–100 nm) and starch granules (1–100 μm), which strongly influence nutrient digestion and absorption [1,2].

These structural considerations are particularly important for starch-rich plant foods. Starch is the major dietary carbohydrate worldwide, contributing approximately 50–70% of the daily energy intake, with cereals and legumes being its principal dietary sources. However, the metabolic effects of these foods depend not only on starch quantity and composition but also on the structural context in which it is embedded. Highly processed starchy foods, including many bakery products, often contain elevated levels of rapidly digestible starch (RDS), partly because milling, refining, and thermal processing can reduce particle size and disrupt native tissue architecture. Regular consumption of such products has been associated with elevated postprandial glycemic and insulinemic responses, which are recognized risk factors for chronic metabolic diseases, including type 2 diabetes mellitus (T2DM) and cardiovascular disease. Repeated exposure to exaggerated postprandial glycemic excursions has also been linked with the development of insulin resistance, impaired glucose homeostasis, and adverse cardiometabolic outcomes. In contrast, preservation of plant structure can slow starch digestion and may contribute to attenuated postprandial metabolic responses [3,4,5,6].

Cereal grains and legume seeds consist of intact plant cells that encapsulate starch, proteins, and lipids within a cellular matrix enclosed by surrounding cell walls. These structures, particularly in mature kernels and seeds, act as physical barriers that restrict hydration, swelling, and digestive enzyme access, particularly in mature kernels and seeds. Starch entrapped within intact cellular structures is classified as resistant starch type 1 (RS1), which is abundant in whole grains, such as wheat, maize, barley, and oats, and in legumes, including lentils, beans, chickpeas, and peas [7]. Consumption of minimally processed cereals and legumes rich in slowly digestible starch (SDS) and resistant starch (RS) has been associated with attenuated postprandial glucose and insulin responses, modulation of gut hormones such as glucagon-like peptide-1 (GLP-1), glucose-dependent insulinotropic polypeptide (GIP), and peptide YY (PYY), enhanced satiety, and improved colonic fermentation through increased short-chain fatty acid (SCFA) production [8,9,10]. These mechanisms may contribute to improved metabolic health and support dietary strategies for the prevention and management of metabolic diseases.

Despite these benefits, cereals and legumes are commonly milled into fine flours with relatively small particle sizes (<100 μm) to produce starch-rich foods, such as bread, pasta, snacks, and other cereal- and legume-based products. Milling reduces particle size and increases the specific surface area available for hydration and enzymatic action. Depending on the milling conditions and extent of tissue disruption, it may also disrupt native cellular architecture and increase the starch exposure to digestive enzymes [11]. Larger flour particles generally have a lower specific surface area-to-volume ratio and may therefore retain a greater proportion of intact cells or cellular aggregates, thereby restricting water penetration and enzyme accessibility [12,13]. Starch may remain more effectively encapsulated within cellular structures, potentially limiting its accessibility during digestion [12]. However, particle size, degree of milling, and cellular integrity should not be considered equivalent structural characteristics. Particle size describes the dimensions of flour particles, whereas cellular integrity refers to the preservation of native cellular architecture and cell walls are preserved. Thus, particles of similar size may differ in terms of cellular preservation, tissue organization, and porosity depending on the milling process and processing history [12,13].

These structural differences are particularly relevant when considering the classification and evaluation of foods from a nutritional perspective. Current dietary guidance emphasizes the consumption of whole grains and the reduction of ultra-processed foods [14,15]. Whole-grain definitions are based primarily on the proportional presence of bran, germ, and endosperm, regardless of whether these fractions remain intact or are reconstituted after processing [16]. Consequently, such definitions provide limited information about the extent to which milling and subsequent processing alter particle size, cellular integrity, and tissue organization within the final food matrix. This distinction may be particularly relevant for understanding differences in starch accessibility and postprandial metabolic responses among products with similar whole-grain content but different structural characteristics.

Despite the growing body of research on food structure, milling, and starch digestibility, the available evidence remains fragmented across studies addressing different aspects of flour particle size, cellular integrity, physicochemical properties, in vitro starch digestion, and postprandial metabolic responses. Particle size is often used as an indicator of structural disruption, although it does not necessarily reflect the extent of cellular preservation. The extent to which milling-induced structural changes translate into physiological responses in humans remains unclear. Evidence from cereals and legumes has rarely been evaluated together while accounting for differences in botanical tissue architecture and processing-induced structural disruption. Therefore, this review examines the effects of milling and flour particle size on the physicochemical and technological properties of cereal- and legume-based foods and evaluates how these structural characteristics relate to in vitro starch and protein digestibility and to acute postprandial and long-term metabolic responses. The review emphasizes the interacting roles of botanical origin, tissue architecture, particle size, and processing conditions in determining carbohydrate functionality and their potential contribution to postprandial metabolic responses while maintaining technological functionality and consumer acceptability.

## 2. Materials and Methods

### Review Approach and Literature Search

This review was conducted as a narrative review because the available evidence comprises studies using diverse methodologies and addressing different but interrelated aspects of food structure, processing, digestibility, and metabolic responses. The literature includes physicochemical and technological characterization studies, in vitro digestion models, acute postprandial and long-term human intervention studies, systematic reviews and meta-analyses. Therefore, a narrative approach was considered appropriate to integrate findings across these different areas of evidence and to critically examine the relationships among botanical origin, tissue and cellular structure, milling, starch digestibility, and metabolic responses. Rather than quantitatively pooling study outcomes, the present review uses the available literature to critically evaluate and integrate evidence across different levels of investigation, with particular attention to the extent to which human evidence supports mechanistic findings from physicochemical and in vitro studies.

Electronic databases, including PubMed, ScienceDirect, and Google Scholar, were searched without date restrictions. The literature search was conducted iteratively and, on a topic-focused basis during review development, with the final search conducted on 5 March 2026. Only articles published in English were considered. The search terms covered the main thematic areas of the review and included “flour particle size,” “intact cells,” “plant cell walls,’’ “milling,” “cereal grains,” “pulses,” “wheat,” “chickpeas,” “beans,” “lentils,” “soybean,” “bread,” “bakery products,” “physicochemical properties,” “texture,” “crumb grain structure,” “starch gelatinization,’’ “water holding capacity,” “in vitro starch digestibility,” “in vitro protein digestibility,” “postprandial glycemic response,” “postprandial insulinemic response,” “gut hormonal response,” “postprandial subjective satiety,” “fullness,” “hunger,” and “desire to eat”.

Literature was selected according to its relevance to the thematic scope of the review and its contribution to understanding the relationships between degree of flour milling, cellular integrity, digestibility, and metabolic responses. Studies were considered relevant when they addressed at least one of these aspects in cereal- or legume-based foods, whereas studies outside the thematic scope, without available full text, or not published in English were excluded. In addition to database searches, relevant articles were identified by manually screening the reference lists of selected publications. Original research was prioritized for specific experimental and human findings, particularly for physiological and metabolic outcomes, while in vitro studies were mainly used for mechanistic interpretation and reviews, meta-analyses, and relevant book chapters for contextualization. Where overlapping evidence was identified, original studies were prioritized for specific findings, and more recent publications were considered, where appropriate, to reflect current evidence.

The final narrative synthesis included approximately 130 publications. The evidence was synthesized qualitatively and critically, considering differences in study design, food composition, processing conditions, structural characteristics, populations, and outcome measures. Findings from reviews and meta-analyses were used for contextualization and were not treated as independent observations alongside the original studies from which the evidence was derived.

## 3. Cellular Architecture and Processing Impact on Cereal and Legume Storage Tissues

Cereals and legumes differ in their anatomical organization, cell wall characteristics, and intracellular architecture, resulting in substantial differences in nutrient bioaccessibility after processing. The endosperm is the principal storage tissue of cereal grains, accounting for approximately 75–80% of the kernel mass. Starch granules are embedded within a protein matrix and surrounded by relatively thin, porous cell walls [11,17,18]. Legume cotyledons constitute approximately 90% of the mature seed and contain starch granules densely packed within a compact protein matrix enclosed by thicker, less permeable cell walls. These structural features restrict starch swelling, limit enzyme penetration, and enhance nutrient encapsulation [2,19,20,21,22].

Cell wall composition plays a central role in determining tissue responses to hydrothermal processing and, consequently, the bioaccessibility of encapsulated macronutrients such as starch and protein [23]. While cooking may cause legume cells to separate from one another, they largely preserve their individual integrity. This behavior is primarily attributed to pectin’s partial solubilization within the middle lamella, which weakens cell-to-cell adhesion without causing extensive cell rupture [24,25,26,27]. In contrast, durum wheat endosperm cells contain little or no pectin in the middle lamella and therefore tend to fracture, exposing intracellular starch to a greater extent [2]. In addition, differences in cell wall permeability contribute to these contrasting behaviors. Cereal endosperm cell walls are generally thinner and more porous, facilitating water penetration and enzyme diffusion, whereas legume cotyledon cell walls are thicker (1–2 μm), denser, and less permeable, limiting hydration, heat transfer, and mass transport [28,29,30].

These structural differences highlight the botanical origin as a major determinant of bioaccessibility and digestive behavior. The extent to which cellular barriers are preserved during processing governs starch accessibility to digestive enzymes and influences postprandial metabolic responses. Compared with cereals, legume tissues retain cellular integrity more effectively, resulting in greater starch encapsulation, reduced enzymatic accessibility, and slower starch digestion.

Table 1 summarizes the comparative structural and compositional characteristics of cereals and legumes relevant to functionality, starch digestibility, and postprandial metabolic responses. Figure 1 summarizes the microstructural differences between legume and cereal endosperm cells and the impact of processing, such as milling, on their cell integrity and starch digestibility.

## 4. Milling as a Structural Modifier of Plant Tissues

Industrial processing modifies the microstructure of plant tissue, thereby influencing nutrient accessibility and digestion [32,33]. Among processing operations, milling is particularly important because it directly alters the physical barriers regulating nutrient release, including seed coats, cell walls, and protein matrices. Consequently, milling affects not only the functionality of flour but also the accessibility of starch and other macronutrients during digestion [34,35].

Milling involves mechanical forces that reduce grains and seeds into flour with specific functional properties. Traditionally, this process has been used to increase nutrient accessibility by disrupting structural barriers and releasing encapsulated starch and protein [20]. However, milling’s nutritional consequences depend not only on particle size reduction but also on the extent to which cellular architecture is preserved. Flours with similar particle sizes may differ in the proportion of intact cells, degree of starch encapsulation, and susceptibility to enzymatic digestion.

Milling can alter the cellular organization and physical state of starch, with the extent of these changes depending on the degree of milling, botanical origin of the raw materials, and the subsequent processing conditions [31,36,37]. Intensive milling may disrupt cell walls and cellular aggregates, whereas more limited structural disruption may preserve a greater proportion of intact cellular structures. These changes can modify the physical accessibility of intracellular components and the physicochemical properties of the resulting flour. Therefore, the degree of flour milling and the resulting particle-size distribution may play an important role in determining the final product properties and, potentially, its metabolic effects [38,39,40]. Table 2 summarizes the structural and nutritional consequences of different milling approaches are summarized in Table 2.

Therefore, milling should be viewed not simply as a particle size reduction process, but as a structural modification that can alter particle architecture, cellular integrity, and the physical accessibility of intracellular components. The implications of these structural changes for flour functionality, digestibility, and postprandial metabolic responses are discussed in the following sections.

Nevertheless, milling should be considered within the broader context of food processing. Subsequent operations, including cooking, baking, steaming, extrusion, fermentation, sprouting, cooling, and reheating, can further modify the structural state of starch and plant tissues and may therefore alter the initial milling effect. These processes can influence starch gelatinization, retrogradation, cell-wall integrity, moisture distribution, and enzyme accessibility, potentially enhancing or attenuating the impact of the initial flour structure. Thus, the present review focuses on milling and flour particle size as primary structural modifiers while considering subsequent processing steps where they substantially modify cellular integrity, starch structure, or digestibility.

Figure 2 summarizes the effects of flour milling degree on particle size, cellular integrity, and the physicochemical and baking properties of cereal- and legume-enriched breads.

## 5. Effects of Milling on the Physicochemical and Techno-Functional Properties of Flour

### 5.1. Effects on the Hydration Properties

Milling-induced changes in the particle architecture and cellular organization can substantially affect the hydration behavior of cereal- and legume-based flours. Therefore, water-holding capacity (WHC) reflects not only particle size but also the extent to which native tissue architecture is preserved. Across cereal and legume flour fractions, including wheat, carob, lentil, bean, pea, and faba bean, coarse fractions generally exhibit greater WHC than finer fractions [12,36,41,42,43,44,45,46,47,48,49]. This behavior can be attributed to the preservation of intact cellular structures, fiber-rich tissues, and intercellular spaces, which facilitate water retention within the plant matrix. In addition, the higher WHC reported for cellular pulse powders compared with conventional pulse flours of similar composition further can confirm the beneficial impact of the preserved cellular architecture [50].

However, the particle size alone does not fully explain the hydration behavior. Comparisons involving relatively narrow particle-size ranges often reveal little difference in WHC, while some studies have even reported lower WHC in coarser fractions [51]. In contrast, intensive milling may increase WHC despite reducing particle size because starch damage enhances water absorption. These findings indicate that hydration reflects not particle size per se, but rather the combined influence of particle architecture, cellular integrity, and starch damage [52,53].

The contribution of tissue structure is also evident in the movement of water within flour particles. Intact cell walls can restrict water penetration and starch swelling, whereas multicellular aggregates may create longer diffusion pathways than isolated cells. This effect may be particularly relevant in legume cotyledons, where dense cellular organization and less permeable cell walls can confer greater resistance to hydration and swelling than in cereal endosperm tissues [12,25,54,55,56].

Although WHC and swelling power (SP) are closely related hydration properties, they can often respond differently to particle size. SP reflects the ability of starch granules to absorb water and expand during heating and is therefore closely linked to gelatinization behavior. Finer flours often exhibit greater SP, as increased surface area and water accessibility can facilitate granule swelling. However, the magnitude of this effect depends on starch damage, cellular integrity, and the flour matrix [45,57,58]. Several cereal flours, including wheat, barley, sorghum, and buckwheat, coarser fractions have been associated with lower swelling and reduced viscosity development [12].

### 5.2. Gelatinization Behavior and Structural Changes During Hydrothermal Processing

Starch gelatinization is a major thermal transition that governs physicochemical properties of flour-based foods. During heating in the presence of water, starch granules swell and lose their crystalline order [59]. In cereal- and legume-based systems, the extent of gelatinization is influenced by particle size and cellular integrity, and water the availability within the food matrix [25].

Across both cereal and legume flours, coarse fractions frequently have been associated with higher gelatinization temperatures than finer fractions, possibly because intact or partially intact cellular structures restrict water penetration and delay starch swelling. In cereals, this effect has been reported in wheat, buckwheat, and rice. However, this relationship is not consistent across all cereal systems. Differences in endosperm organization, starch content, and the extent of cellular disruption can influence thermal behavior, indicating that particle size alone is not a reliable predictor of gelatinization characteristics [36,49,58,60,61,62].

The influence of cellular integrity is particularly evident in legumes. Compared with cereal endosperm tissues, legume cotyledons possess relatively thicker, less permeable cell walls and dense protein matrices that restrict water uptake, starch swelling, and amylose leaching [19,54]. Intact cells from chickpea, pea, mung bean, and kidney bean often remain only partially gelatinized after hydrothermal treatment, retaining birefringence and characteristic Maltese crosses under polarized light [19,63]. These observations indicate that starch encapsulation within intact legume cells can persist in isolates starch systems despite conditions that would normally promote extensive gelatinization.

The thermal behavior of legume flours reflects this structural resistance. Preservation of cellular architecture has been associated with reduced starch swelling, higher gelatinization temperatures, and, in some cases, greater energy requirements for structural disruption than isolated starch or disrupted flour particles [19]. However, responses are not always consistent across botanical sources. While coarser chickpea and lentil fractions often exhibit higher gelatinization temperatures, studies in pea, lentil, and faba bean flours have reported limited or inconsistent particle-size effects [46,48,49,64]. These contrasting findings indicate that gelatinization behavior in legumes is determined not only by particle size but also by the extent of starch encapsulation, cellular preservation, and intrinsic starch properties.

Studies of cellular powders enriched in intact plant cells further highlight the role of structural preservation in the starch thermal behavior. These materials often exhibit lower gelatinization enthalpy and narrower thermal transition ranges, reflecting the moderating effect of cellular architecture on starch phase transitions [24]. Similarly, differences between flours containing predominantly intact or ruptured cells are frequently smaller than expected based on particle size alone. Overall, the available evidence indicates that starch encapsulation within intact cellular matrices can attenuate thermal disorganization, supporting the view that structural integrity governs gelatinization behavior more than by particle size per se [64].

### 5.3. Dough and Bread Properties

The incorporation of coarse cereal or legume flour fractions into starchy foods can present technological challenges, as increases in particle size may affect dough rheology, gas retention, crumb structure, texture, and overall product quality. Bread has been the most extensively studied model system, with studies generally reporting lower loaf volume, reduced porosity, denser crumb structure, and greater hardness and chewiness as wheat flour particle size increases [8,41,65]. These effects have also been observed in breads prepared from cracked wheat and coarse semolina, although the contribution of particle size cannot always be separated from differences in grain structure, composition, and processing conditions. Evidence from wheat breads further indicates that very coarse fractions (>1000 μm) can compromise product quality more severely than moderately coarse flours, highlighting the existence of a practical threshold beyond which further increases in particle size provide limited technological benefit [62].

Research on the effects of legume particle size and cellular integrity on bread quality remains relatively limited. Nevertheless, studies examining these factors have reported broadly similar trends. Incorporation of bean- or chickpea-based ingredients, particularly in the form of coarse flours or cellular powders, has generally been associated with reduced loaf volume and increased crumb hardness and chewiness, reflecting the disruption of dough structure by larger, structurally intact particles [5,48,66]. The observed differences may reflect the combined influence of particle size, cellular structure, ingredient composition, and the level of legume incorporation on dough development and bread structure. In some cases, these effects become more pronounced at higher legume incorporation levels, resulting in denser crumb structures and reduced porosity. However, sensory responses do not always mirror instrumental quality measurements, and several studies have reported little or no adverse effect on overall consumer acceptability despite measurable changes in texture and loaf characteristics [7]. These findings suggest that while maintaining acceptable product quality, structurally preserved legume ingredients can be incorporated into bread formulations at moderate levels while maintaining acceptable product quality, although further research is needed to define optimal particle-size ranges and formulation strategies.

## 6. Effects of Flour Particle Size, Cellular Integrity and Botanical Origin on In Vitro Digestibility

Flour particle size is a major determinant of starch digestibility and is closely linked to plant tissue structural integrity. The effects of particle size and cellular integrity on starch digestibility have been extensively investigated using in vitro digestion models. Overall, finer particle fractions and greater disruption of cellular structures are often associated with faster starch hydrolysis, whereas the preservation of intact cellular structures may delay enzyme access and starch breakdown [29,61,63,67]. However, the magnitude and consistency of these effects vary according to botanical origin, food matrix, degree of milling, and subsequent processing conditions.

The importance of particle size has been incorporated into kinetic models of starch digestion, highlighting its predictive value in determining both the rate and extent of carbohydrate hydrolysis [68]. However, its physiological relevance cannot be interpreted independently of the structural changes accompanying milling. The effects of particle size depend on the extent to which milling disrupts native cellular architecture, alters starch–protein interactions, and modifies water penetration during subsequent processing and digestion [61,67]. Accordingly, flour particle size should be viewed not only as a geometric determinant of enzyme accessibility, but also as a practical marker of structural preservation.

### 6.1. Starch Digestibility

#### 6.1.1. Cereals and Legumes

Ιsolated intact cereal cells provide important mechanistic insight into the role of tissue structure in regulating starch digestion. Starch encapsulated within intact endosperm cells of wheat and sorghum is hydrolyzed at significantly lower rates and to a lesser extent than corresponding finely milled flours or isolated starch preparations [69]. In addition, although hydrothermal treatment can increase starch digestibility by promoting gelatinization and partial softening of cellular barriers, substantial differences between intact and disrupted systems often remain. For example, heating buckwheat cells at 70 °C more than doubled the digestibility of starch after prolonging in vitro digestion relative to untreated cells. Yet hydrolysis remained markedly lower than in flour systems, indicating that the preserved cell wall integrity and limited permeability to α-amylase continue to restrict enzyme access [70].

The inverse relationship between particle size and starch digestibility has also been demonstrated across a range of cereal flours. Porridges prepared from coarse wheat flour (1950 μm) exhibited up to 50% lower total sugar release during in vitro digestion than porridges made from fine flour (<110 μm) [71]. Similarly, wheat semolina (750–800 μm) showed up to 40% lower digestion rates than conventional wheat flour (85 μm), which may be attributed to the preservation of cell clusters and the restriction of amylase diffusion [12]. Additional evidence indicates that increasing particle size can reduce both the rate and extent of starch hydrolysis. For example, coarse farina fractions (999 μm) displayed a 76% lower hydrolysis rate than conventional flour when modelled using first-order digestion kinetics [36]. Comparable trends have been reported in coarse durum wheat fractions (>1290 μm), where intact cells effectively encapsulated starch and delayed digestion [29,61].

Similar patterns have been observed in other cereals, although the magnitude of the effect appears species-dependent. Larger particle sizes in sorghum and barley are associated with reduced digestibility, but differences in cell wall permeability influence the extent of protection. In sorghum, particles >1000 μm may remain partially undigested even after 24 h of in vitro digestion, whereas barley starch is largely hydrolyzed within the same period [72]. Likewise, in maize, coarse flour fractions (130–180 μm) exhibit slower starch digestion than fine fractions (<80 μm), likely due to compact tissue structure, dense starch granule packing, and retention of intact cell walls [59].

Legume seeds generally show greater resistance to starch digestion than cereal grains. As in cereals, disruption of the native tissue architecture through fine milling substantially increases the bioaccessibility of starch. The reduction in particle size decreases the RS1 proportion, exposes encapsulated starch, and accelerates hydrolysis [48]. In legumes, however, the effects of milling may be more pronounced than in cereals because the intact cotyledon cell wall is often a stronger diffusion barrier and remains functional even after hydrothermal treatment. Studies from chickpea systems highlight the importance of the preservation of cellular integrity. Precooked cellular powders retaining intact chickpea cells exhibited markedly lower starch digestibility (<40%) than conventionally milled flours (~80%) after 90 min of the in vitro digestion [24]. Similar findings have been reported in model porridges containing pre-gelatinized flour, where samples enriched in intact cells showed lower hydrolysis rates than flours composed predominantly of ruptured cells [10]. These results indicate that even after thermal processing, cellular encapsulation can remain effective.

Comparable trends have been observed for other legumes. In cooked beans, intact seeds digest at lower rates than finely milled flour or isolated starch preparations [54]. In dry-milled bean flour, increasing particle size reduced the starch hydrolysis rate, while coarse fractions (710–1190 μm) contained approximately twice the amount of RS and 3.5-fold more insoluble non-starch polysaccharides than finer fractions [44]. In faba bean, increasing the particle size from 14 μm to 0.5 and 1 mm reduced digestible starch and increased the RS content [47]. Similarly, increasing the particle size of lentil flour from 28 μm to 1042 μm progressively reduced glucose release during simulated digestion [73].

In addition, strong particle-size effects have also been demonstrated in pea ingredients. In hydrothermally processed pea flour fractions, coarse particles (>500 μm and particularly >1000 μm) exhibited only ~25% starch hydrolysis during the first hour of digestion, compared with finely milled flour or isolated starch controls (>80%) [28]. Such findings highlight the role of structural preservation in delaying starch digestion, even after the ingredients have undergone prior thermal treatment.

Similar effects have been demonstrated in composite legume foods. Hummus containing intact chickpea cells exhibited slower digestion than formulations in which cells were mechanically ruptured. Microscopy confirmed greater preservation of cellular integrity after digestion, while rheological analysis indicated higher storage modulus despite fracture at lower deformation levels, with both structure and texture potentially influencing the digestive behavior [74].

The effects of the initial flour structure are not necessarily preserved throughout processing. Overall, the available in vitro evidence generally shows an association between larger particle size, greater preservation of cellular structure, and slower starch hydrolysis across cereal- and legume-based systems. However, the relationship is not strictly linear, as botanical source, cell wall composition, protein matrix density, degree of gelatinization, and prior thermal or mechanical processing may affect the rate and extent of starch hydrolysis. Hydrothermal treatments, such as cooking and baking, can modify or partially override milling differences by altering starch gelatinization, cell-wall integrity, and matrix organization. Therefore, the relationship between flour particle size and starch digestibility should be interpreted in the context of the food’s complete processing history.

#### 6.1.2. Bread, Pasta, and Biscuits

Bakery products and other starch-rich foods are major contributors to dietary carbohydrate intake and postprandial glycemic response. Although flour particle size and cellular integrity preservation are associated with differences in starch digestibility in isolated cereal and legume flours, the translation of these effects to real food systems is more complex. The incorporation of coarse flour fractions into products such as bread, biscuits, and pasta creates a new food matrix shaped by mixing, proofing, extrusion, and baking processes, all of which can modify starch organization, cell wall integrity, and enzyme accessibility [37,67]. Consequently, the physiological relevance of particle size may be better evaluated at the level of the final food matrix rather than the raw flour alone.

Hydrothermal processing, particularly baking, is a critical determinant of digestibility because the combined effects of heat and moisture alter plant tissue microstructure and the structural barriers functionality. Rapid moisture evaporation during baking may increase tissue brittleness, whereas lower water availability relative to wet heating can permit partial preservation of native cellular structures [70]. Thus, starch bioaccessibility is governed by the interaction between particle size, cell wall integrity, protein network formation, and thermal history. Table 3 presents the relevant studies that investigated the impact of plant tissue structure (cereal vs. legumes) and the industrial flour milling on the in vitro digestibility.

Several studies in commercial and model breads indicate that inclusion of coarse cereal fractions can substantially reduce in vitro starch digestion when structural integrity is sufficiently preserved. In commercial breads, the incorporation of >65% coarse wheat fractions (>1000 μm) significantly reduced starch hydrolysis, although sample homogenization prior to digestion increased the hydrolysis rate, confirming the importance of intact structural barriers in limiting enzyme access [75]. Similarly, the inclusion of 80% intact wheat kernels in white wheat bread significantly reduced starch hydrolysis relative to conventional bread formulations [76].

Wholegrain breads produced from larger flour particles also show slower and less extensive starch digestion. Increasing the flour particle size of whole wheat from 199 to 1325 μm reduced both the rate and extent of starch hydrolysis, likely due to denser fiber networks, lower specific surface area, and reduced loaf expansion, which together restricted enzyme penetration [41]. The inclusion of 75% kibbled purple wheat also significantly reduced the rate of glucose release [77]. In addition, the replacement of roller-milled wheat flour (<150 μm) with coarse wheat fractions (>1680 μm) or blends containing intact kernels significantly reduced the estimated glycemic index and RDS, while increasing the RS1 content. Furthermore, incorporation of 30% intact kernels increased RS2, which accounted for approximately 35% of total RS [78].

Comparable effects have been reported for other cereal products. Rice bread produced from coarse rice flour fractions showed lower RDS and higher SDS than breads prepared from finer flour [79]. In biscuits, incorporation of wheat particles (~1000 μm) significantly reduced starch digestibility and increased SDS relative to conventional fine flour controls [36]. Coarser maize flour similarly reduced the rate and extent of starch hydrolysis in extruded corn pasta, confirming that milling intensity remains relevant even after extrusion processing [80]. In another study, reducing the degree of grain milling in oat, wheat, and maize scones led to the preservation of grain integrity and a decrease in in vitro starch digestibility. Among these, whole maize scones exhibited the lowest rates and extents of amylolysis, followed by those made with whole wheat, whereas whole oats scones showed the highest values [81].

However, not all studies have reported beneficial effects of coarse particles. Replacement of white wheat flour with coarse farina (705 μm) up to 80% did not significantly alter starch hydrolysis [12]. Likewise, wheat fractions of medium (1000–1800 μm), and large particle size (>1800 μm) failed to retain their lower flour-level digestibility after breadmaking [13]. These discrepancies may reflect the structural degradation of cell wall polysaccharides during mixing, fermentation, and baking, which increases porosity and starch accessibility despite the initial preservation of particle integrity.

Compared with cereals, legume ingredients often produce stronger digestibility-lowering effects. Accordingly, incorporation of coarse legume fractions or intact-cell ingredients into starch-rich foods can substantially reduce starch bioaccessibility and in vitro hydrolysis, although the magnitude of this effect is matrix-dependent.

The incorporation of 20% cannellini bean flour containing intact cells (~200 μm) into bread reduced the rapidly available glucose by approximately 50% compared with that of finely milled legume flour (<100 μm) [48]. Similarly, the replacement of wheat flour with 75% of a soya/purple wheat blend (>2800 μm) significantly reduced glucose release after 120 min of in vitro digestion, with the soy component further attenuating starch hydrolysis due to its higher protein content [77]. Another study found that substitution of wheat flour with chickpea flour containing intact cells dose-dependently reduced starch hydrolysis, with greater reductions observed at 60% replacement (~75% vs. ~41%). Microscopy confirmed that the starch entrapped within the chickpea cells remained partially protected even after baking [7]. Biscuits and crackers appear to be particularly suitable matrices for preserving the structural functionality of legume particles, as their relatively low-moisture processing conditions may limit complete starch gelatinization and excessive cell wall disruption. In savory biscuits, replacing of wheat flour with 25–100% chickpea flour significantly reduced starch digestibility, with 50% substitution producing the lowest hydrolysis index, indicating that maximal functionality may not necessarily occur at complete flour replacement [82]. Biscuits prepared from black soybean flour fractions showed a progressive reduction in RDS and increases in SDS and RS with increasing particle size, supporting the view that reduced surface area and limited enzyme penetration are key mechanisms underlying slower starch hydrolysis [83]. This pattern was also observed in, crackers prepared from very coarse chickpea particles (2000–7000 μm), which exhibited lower starch hydrolysis than those made from finer particles (<2000 μm), suggesting that coarse particle incorporation may extend beyond bread systems [84].

Pasta products also appear to respond to structural modification through legume incorporation, although the mechanisms may differ from bakery products, as protein-network formation strongly contributes to matrix density. The replacement of 30% of conventional lentil flour with flour containing intact plant cells, or with whole pre-cooked lentil powder, significantly reduced starch digestion after 300 min of simulated gastrointestinal digestion [85]. The strongest effect was observed with isolated intact cells, followed by whole pre-cooked powder, whereas conventional flour showed the highest hydrolysis. These findings suggest that both cellular encapsulation and denser protein–starch matrix can restrict enzymatic accessibility in pasta systems.

### 6.2. Protein Digestibility

Although this review primarily focuses on starch digestibility, the structural characteristics of cereal- and legume-based foods may also influence the accessibility and digestion of plant proteins. Proteins derived from cereals, legumes, and nuts are increasingly recognized for their nutritional value, lower environmental impact, and potential health benefits [86]. However, compared with starch, relatively less attention has been given to the role of particle size, cellular architecture, and cell-wall integrity in determining protein bioaccessibility and digestibility. Therefore, whether structural features that limit starch accessibility, such as larger particle size and preservation of cellular integrity, exert comparable effects on protein digestion, or whether protein digestibility responds differently to changes in food structure and processing. The available evidence is more limited and less consistent than that for starch and may depend on botanical origin, protein localization, matrix composition, and processing conditions. Accordingly, this section examines the relationship between particle size, cellular integrity, and protein digestibility as a complementary aspect of the structural effects of milling.

In general, reduction in particle size tends to increase in vitro protein digestibility by increasing surface area and exposing intracellular proteins to digestive enzymes. In durum wheat porridges, finer particles (110 μm) produced greater protein release (62.6%) than coarser fractions (1440–1950 μm; 33.4–35.1%), together with faster release during the duodenal phase [71]. A similar pattern was observed, in cooked bean flour, with smaller particles (<74 μm) showing greater and faster than larger fractions (74–500 μm) [87]. These effects are commonly attributed to the higher surface-area-to-volume ratio of smaller particles and the presence of intact cellular aggregates in coarser fractions, which act as physical barriers to protease access [83,84,88].

**Table 3:** Effect of plant tissue structure and industrial flour milling on in vitro digestibility.

Plant Tissue Type	Tissue Intactness Level/Milling Fraction	Processing Treatment	In Vitro Digestion Model	Control Sample	In Vitro Digestibility	Structure During/After In Vitro Digestion	Ref.
Barley Sorghum	Fractions (mm) of 0.250, 0.5, 1.0, 1.7, 2.8	Hammer milling, Sieving	Three-step closed-system enzymatic hydrolysis (oral, stomach, small intestine phases)	Unsieved barley and Sorghum	↑ Starch hydrolysis with ↓ particle size (complete digestion in 1 mm barley; incomplete in sorghum)	Smaller particles: starch depleted Larger particles: residual starch granules	[72]
Chickpeas	Hummus with the following particles size: Intact chickpea cells (ICC) Ruptured chickpea cells (RCC)	Dry milling, crushing, grounding, hydrothermal process and blending (for the RCC sample)	In vitro enzymatic hydrolysis	No external control	↓ Rate of digestion for ICC hummus (vs. RCC hummus) ↓ Extent of digestion of ICC (vs. RCC hummus)	Visible intact cotyledon cells containing intracellular starch in ICC hummus Extensive amylolysis and loss of cellular integrity in RCC hummus	[74]
Chickpeas	Breads with intact cell powder (ICP) 30% ICP bread 60% ICP bread	Isolation of ICP, hydrothermal processing, wet sieving, drying	In vitro enzymatic hydrolysis	0% ICP bread	Starch digestion rate and extent 0% ICP > 30% ICP > 60% ICP	Starch encapsulated within the ICP retained its resistance following bread baking	[7]
Navy beans	Whole navy beans Milled bean flour Damaged cotyledon cells	Soaking (1 h), Autoclaving (15 min, 121 °C) Sample forms: Whole beans, flour (milled, sieved), damaged cells (French press)	Two-phase static in vitro gastrointestinal digestion	Isolated bean starch	Extent of hydrolysis in cooked samples bean starch = bean flour (85–90%) > whole beans (60%) (120 min) ↑ After mechanical damage (* vs. intact cotyledon cells) ↓ After storage of cooked beans (40–45%)	Cotyledon cell’s structure maintained Slightly shrunken appearance and surface wrinkles that increase with time	[54]
Chickpea Red kidney beans Mung beans Peas	Intact cells Broken cells	Intact cell isolation: Soaking in water, manual dehulling, thermal treatment (60 min, 95 °C), mashing (mortar and pestle), wet sieving (53 to 150 µm fraction)	Dynamic in vitro rat stomach duodenum model (DIVRSD)	No external control	Hydrolysis extent Broken cells (45–50%) > Intact cells (2–3%) Starch hydrolysis extent and starch hydrolysis rate mung bean > pea > chickpea > red kidney bean	Retention of cellular integrity of intact cells	[25]
Wheat Sorghum	Intact cellular cells Broken cells Flour	Isolation of intact cells: Pearl de-branning of wheat, dry milling, wet sieving, hydrothermal process (30 min, 70 °Cand 75 °C respectively for wheat and sorghum)	In vitro enzymatic hydrolysis	Isolated starch	Uncooked samples Starch digestion extent starch > broken cells > flour > intact cells in both wheat and sorghum Cooked samples ↑ Starch digestion extent compared to the uncooked samples Slightly ↑ starch digestion extent in the cooked intact cells compared to uncooked intact cells in both sorghum and wheat	Cellular integrity retained (independent of processing conditions)	[69]
Beans	Cannellini bean flour (CBF) Cannellini Bean Flour Fraction Rich In fractured cells (CBFC) Cannellini bean flour fraction rich in intact cells (CBIC)	F1 bread (20% cannellini bean flour) F2 bread (20% cannellini bean flour fraction rich in fractured cells) F3 bread (20% cannellini bean flour fraction rich in intact cells)	In vitro enzymatic hydrolysis	White rice flour bread (RB)	Starch digestion rate index: RB > F1 > F2 > F3 Rapidly available glucose: RB > F2 > F1 > F3	Not post-digestion structural analysis	[48]
Tartary buckwheat (TB) Common buckwheat (CB)	Intact endosperm cells Light flour Broken cells	Dry milling, dry sifting, magnetic stirring, wet sieving, hydrothermal process (30 min, 76 °C and 75 °C respectively for TB and CB respectively)	In vitro enzymatic hydrolysis	Isolated starch	Starch hydrolysis extent: Uncooked TB and CB fractions isolated starch > partially broken cells > light flour > intact cells Cooked TB and CB fractions isolated starch > broken cells > light flour > intact cells	Not post-digestion structural analysis	[89]
Rice	Coarse particles Fine particles	Coarse rice bread (CRB) Fine rice bread (FRB)	In vitro enzymatic hydrolysis	White bread (WB)	↔ Extent of starch hydrolysis extent ↑ Rate of digestion of CRB (* vs. FRB)	Not post-digestion structural analysis	[79]
Chickpea	Resistant starch-enriched chickpea powder (RSF) in the following forms 0% RSF enriched biscuit 25% RSF enriched biscuit 50% RSF enriched biscuit 75% RSF enriched biscuit 100% RSF enriched biscuit	Hydrothermal treatment to isolate the intact cells	In vitro enzymatic hydrolysis	Wheat and chickpea flours in boiled form	50% RSF biscuit had the lowest starch digestibility, hydrolysis index and extent of hydrolysis after 90 min	Cellular integrity of RSF particles in biscuits retained	[82]
Chickpea Red kidney beans Mung beans Peas	Intact cells Broken cells	intact cell isolation: soaking in water, manual dehulling hydrothermal treatment (60 min, 60 °C or 95 °C), mashing (mortar and pestle), wet sieving (53 to 150 µm fraction)	In vitro enzymatic hydrolysis	Isolated starch from the same legumes	Hydrolysis rate (under stirring and non-stirring conditions) Broken (95 °C) > isolated starch (60 °C) > intact cells	Retention of cellular integrity of intact cells (in both 60 °C and 90 °C)	[63]
Adzuki bean Chickpea Lentil Lima bean	Intact cells	Isolation of cotyledon cells: Soaking in HCl (24 h, 20 °C), manual dehulling and separation of seeds into cotyledons, soaking in NaOH (24 h, 20 °C), mashing (mortar and pestle), wet sieving (53 to 150 µm fraction), dehydration with graded ethanol series, freeze drying	Two-phase static in vitro gastrointestinal digestion	Isolated starch from the same legumes	Hydrolysis rate and extent isolated starch granules > isolated cotyledon cells	Unaffected cells through gastric digestion Change in small intestinal phase	[19]
Chickpea Durum wheat	<0.21, 0.38, 0.55, 0.73, 1.02, 1.29, 1.55, 1.85, 2.58	De-branning of wheat grains, manual dehulling of chickpeas, roller milling, sieving	In vitro enzymatic hydrolysis	Chickpea and wheat isolated starches	↓ Starch digestion extent with ↑ of particle size	Not post-digestion structural analysis	[61]
Durum wheat	Durum wheat flour in porridge made with smooth or coarse flour	Roller-milled dehulled/de-branned, cooking under standardized conditions	In vitro enzymatic hydrolysis	Gelatinized isolated starch	↓ Hydrolysis rate and extent with increasing particle size (reference > smooth > coarse porridge)	Not post-digestion structural analysis	[29]
Wild-type pea (WT) r-mutant pea (rrRbRb)	Fractions of <0.25, >0.25, >0.5, >1 mm	Soaking, manual dehulling, drying, milling, sieving	In vitro enzymatic hydrolysis	Isolated starch from both legumes	↔ Starch digestibility between fractions of the same source ↓ Starch digestion extent with increasing particle size ↓ Starch digestibility of r-mutant isolated starch compared to WT ↔ Starch digestion extent between two sources for fractions > 1 mm	Not post-digestion structural analysis	[28]
Durum wheat Chickpea	Chickpea and durum wheat with fractions of <0.21, 0.38, 0.55, 1.85 mm Chickpea and durum wheat in intact and homogenized form	Soaking overnight, manual dehulling of chickpeas, de-branning of durum wheat, roller milling, sieving	Combination of dynamic gastric (DGM) and static duodenal (SDM) in vitro model	Chickpea and durum wheat starch	↓ Starch hydrolysis rate and extent with increasing particle size in both chickpea and wheat particles. ↑ Digestion extent in durum wheat (vs. chickpeas) Hydrolysis extent Homogenized > Intact (final extent NS in wheat)	Chickpea cells remained intact and separated Wheat cells ruptured.	[24]
Wheat Oats Maize	Wheat: Fine flour, Whole, Cracked, Coarse grains Maize: Fine flour, Cracked, Whole grains Oats: Fine flour, Rolled oats, Whole grains	Baked products of wheat, maize and oats	In vitro enzymatic hydrolysis	No external control	↓ Starch digestion with increasing particle size in all baked samples	Not post-digestion structural analysis	[81]
Smooth Pea	Intact cells (IC) Broken cells (BC) Slightly damaged cells (SDC) Highly damaged cells (HDC)	Isolation of intact pea cells: Soaking in NaHCO_3_ and Na_2_CO_3_ (4 °C), manual removal of outer layers, thermal treatment (excess distilled water, for 1 h, at 60 °C), mashing (mortar and pestle), sieving, freeze drying (48 h), Broken cells: Shear mixing force, starch isolation	Static in vitro gastrointestinal digestion (INFOGEST 2.0)	Isolated starch	Starch digestion extent isolated starch > BC > HDC > SDC > IC	IC retained the highest cell wall integrity and molecular weight	[90]
Durum wheat endosperm	Coarse farina Fine farina Flour	Roller milling, sieving, hydrothermal treatment (hydrothermally treated samples) or direct storing at −20 °C (regular samples)	In vitro enzymatic hydrolysis	No external control	Regular Samples ↔ Starch digestion extent ↑ Starch digestion rate in flour compared to fine and coarse farina Hydrothermally treated samples ↔ Starch digestion extent ↑ Starch digestion rate in flour and fine farina compared to coarse farina	Regular samples ↑ Number of cells with intact call walls in coarse farina than flour Hydrothermally treated samples Maintenance of cell wall intactness of clustered cells in coarse farina	[12]
Garbanzo bean seeds Pinto bean seeds	Intact cells	Isolation of cotyledon cells: Soaking in water, dehulling, thermal treatment (60 min, 60 °C), mashing (mortar and pestle), wet sieving (100–250 μm fraction), freeze drying	In vitro enzymatic hydrolysis	Isolated starch from the pulses	↑ Gradual starch digestion rate and extent with temperature in intact cells ↓ Starch digestion rate and extent for isolated pulse cells compared to isolated starch	Untreated isolated pulse cells retained granular structure while incubation in higher temperatures gradually increased susceptibility	[91]
Wheat	Coarse flour Medium flour Fine flour Control flour	Coarse bread Medium bread Fine bread	Three-phase oral–gastro-intestinal in vitro digestion	White wheat bread	Starch rate of digestion: coarse < medium bread < white wheat bread < fine bread Extent of digestion: fine > control > medium > coarse	Not post-digestion structural analysis	[41]
Navy beans	Raw navy bean flour and baked dough (Coarse, regular, very fine, fine, super fine particle size)	Dry milling (pre-ground with hammer mill, milled with pin mill), baking, dough formation (cooked bean flour + water), dough baking	Static in vitro gastrointestinal digestion	No external control	↓ Digestion rate and ↑ cumulative glucose release with increasing particle size in both raw and baked flours	Not post-digestion structural analysis	[44]
Durum wheat	Wheat porridges: A:0.11 mm (flour) B: 0.38 mm (small) C: 1.01 (semolina) D: 1.44 (medium) E: 1.95 (large)	Roller milling debraning of durum wheat grain, thermal treatment (5 min, 85 °C), adding cold water, reheating (5 min, 85 °C), boiling (5 min), cooling (15 min)	Oral digestion, dynamic gastric model (DGM) followed by a simulated duodenal digestion	No external control	↑ Starch degradation in porridge A (* vs. E), ↔ in C, D and E ↓Glucose release with increasing particle size	Not post-digestion structural analysis	[71]
Chickpea	Non-precooked open cotyledon cell flour (NC-O) Precooked open cotyledon cell flour (PC-O) Pre-cooked individual cotyledon cell flour (PC-I)	NC-O: Soaking, dehulling, mechanical disintegration, lyophilization (24 h), sieving (500 μm mesh) PC-I: overnight soaking, cooking (60 min, 95 °C), dehulling, mechanical disintegration, lyophilization, sieving PC-O: ball milling PC-I	Static in vitro intestinal digestion (INFOGEST 2.0)	No external control	↓ Starch digestion rate of PC-I (* vs. NC-O, PC-O) ↔ Starch digestion extent	Not post-digestion structural analysis	[10]
Beans	Isolated cells Cell clusters	Soaking in water, thermal treatments: high temperature (HT), high temperature high pressure (HPHT), high pressure (HP), dehulling, mechanical disintegration, particle size distribution or lyophilization	In vitro enzymatic hydrolysis	Fully gelatinized free starch granules	Hydrolysis extent gelatinized starch > intact individual cells > cluster cells (↑ with processing severity)	Not post-digestion structural analysis	[27]
Beans	Intact cotyledon cells	Soaking (16 h, 25 °C), thermal processing (30, 60, 90, 120 and 180 min at 95 °C), dehulling, mechanical disintegration	In vitro enzymatic hydrolysis	No external control	↑ Hydrolysis rate and extent with time (180 min > 30 min; intermediate NS)	Cellular integrity retained (independent of processing and digestion time) Progressive intracellular starch hydrolysis (180 min > 90 min > 30 min)	[26]
Maize	Hard and soft flour: Coarse, medium, fine	De-branning and removal of germ, roller-milling, sieving, re-milling (hammer mill), sieving	In vitro enzymatic hydrolysis	No external control	↓ Hydrolysis extent with ↑ particle size (strong in native form; reduced after hydrothermal processing)	Not post-digestion structural analysis	[59]
Red kidney bean	Intact cells (ICC) Mechanically damaged cells (MDC) Enzymatically damaged cells (EDC)	Isolation of cotyledon cells: Soaking in water, dehulling, thermal treatment (60 min, 95 °C), mashing (mortar and pestle), wet sieving	Two-phase static model: Gastric and Intestinal Phase	Isolated starch	Hydrolysis extent ↔ for all samples (6 h) Hydrolysis rate MDC (50%) > EDC (30%) > ICC (less than 10%) (10 min)	ICC and EDC retained their integrity Mechanically damaged cells were ruptured Cytoplasmic matrix retains its compact organization.	[21]
Soy Wheat Rye Linseed	Intact whole grain range (% on dry weight basis): Rye bread 65% All the other test breads < 30%	Gluten-free bread Wholemeal bread Super thick bread Mixed grain Grain plus Light grains Rye bread Soy–linseed/Low carb Soy–linseed	In vitro enzymatic hydrolysis	White wheat bread (no >1 mm particles)	Hydrolysis extent intact > homogenized (effect only at coarse particle content > 65%) ↓ GGE with high intact grain content (18 vs. 34)	Not post-digestion structural analysis	[75]
Purple wheat Soya	Kibbled flours	Kibbled purple wheat bread (PB) Kibbled soya–purple wheat bread (SPB)	In vitro enzymatic hydrolysis	White bread (WB)	Starch digestion rate and extent: ↔ WB (vs. homogenizing form) ↓ RB, SPB (vs. homogenizing form) ↑ Relative glycemic potency of homogenized (vs. intact forms of breads) Relative glycemic potency in both intact and homogenized forms: WB > RB > SPB	Not post-digestion structural analysis	[77]
Chickpea	Chickpea puree and cracker: Fine puree Coarse puree Fine cracker Coarse cracker	Overnight soaking of dry chickpeas, boiling, milling, sieving	Dynamic static in vitro gastric-small intestinal digestion	No external control	Release of reducing sugars: fine cracker > fine puree > coarse puree > coarse cracker ↑ Starch hydrolysis in fine cracker	Not post-digestion structural analysis	[84]
White beans Red lentils Yellow peas	Raw seeds: White kidney beans Red lentils Yellow peas	Soaking + Cooking (C) Cooking + Blending (C + B) Milling + Blending (M + B)	In vitro enzymatic hydrolysis	Rice starch Isolated starch from the legumes	Starch digestion M + B > C + B > C	Soaked cells remain intact with ungelatinized starch. S + C had gelatinized starch	[56]

The studies summarized in this table used heterogeneous in vitro digestion protocols, including differences in digestion models, enzymatic conditions, digestion phases, sampling times, controls and outcome definitions. Therefore, hydrolysis values and digestibility outcomes are presented to summarize the findings of individual studies and should not be interpreted as directly comparable across studies. Abbreviations: * indicates a statistically significant difference between the treatments (*p* < 0.05). NS, non-significant. Comparable trends have been reported across other plant materials and food products. Protein digestibility in sourdough wheat bread was negatively correlated with particle size (r = −0.854), whereas finer fractions were positively associated with higher digestibility (r = 0.703) [92]. In sorghum flour, particle size reduction improved both starch and protein digestibility, supporting the view that milling enhances enzyme accessibility to multiple macronutrients [93]. Similarly, chickpea-based foods such as crackers and purées showed greater protein hydrolysis when prepared from finer particles [84].

However, the effect of particle size strongly depends on crop type and matrix microstructure. For example, finer barley flour showed slightly higher protein digestibility than coarser fractions (77.1% vs. 73.0%), whereas no significant effect of particle size was observed in oat, pea, and lentil flours [94]. Similar findings have been reported for faba bean flour, where increasing particle size from 140 to 1000 μm did not significantly alter protein digestibility, which remained relatively constant (~75.7–76%) [47]. These inconsistencies suggest that the influence of particle size can be moderated by botanical origin, protein localization, and cell-wall characteristics.

Food processing conditions also play a major role, as technological treatments can alter food microstructure and protein accessibility. Structural changes induced during mixing and baking may weaken or override the effects of initial flour particle size [13]. In legume ingredients with preserved cellular structure, thermal treatment can markedly influence digestibility. Flour produced from whole pre-cooked lentils retained much of its structural integrity after short cooking and exhibited lower proteolysis than raw milled flour or isolated cotyledon cells, owing to the persistence of intact cellular aggregates that limited enzyme access [95]. Similarly, boiling navy bean flour resulted in higher protein digestibility than baking or roasting [87].

Milling technology may also influence outcomes through its effects on particle microstructure. In cowpea flour, the milling method had only modest effects overall, although hammer-milled samples showed slightly greater digestibility than cryo-milled samples. Moreover, particle size affected protein release in cryo-milled but not hammer-milled samples, highlighting the potential role of milling technology in altering internal particle structure and subsequent enzymatic accessibility [96].

Evidence from composite foods further highlights the role of cellular encapsulation. Replacement of refined wheat flour with intact chickpea cell powder in bread reduced early gastric protein release, as proteins remained entrapped within intact plant cells. However, a substantial proportion of protein had been hydrolyzed by the end of the duodenal phase, indicating that intact cellular structures delay, rather than completely prevent, digestion. Confocal microscopy confirmed that proteins visible within intact cells during oral and gastric phases were no longer detectable after prolonged intestinal digestion, demonstrating that the protective effect of cellular structure is dynamic but ultimately transient [7,97,98].

## 7. Effect of Particle Size and Structural Integrity of Plant Tissues on Postprandial Glycemic and Hormonal Responses

In vitro digestion studies have consistently demonstrated that increasing the particle size in cereal- and legume-based foods reduces the rate and extent of starch hydrolysis. However, the physiological relevance of these findings needs to be evaluated in vivo, where postprandial responses are additionally influenced by factors beyond starch digestion, such as mastication, gastric emptying, hormonal regulation, insulin sensitivity, and inter-individual variability. Consistent with the FAO/WHO (1998), smaller flour particle size is generally associated with higher glycemic responses, primarily due to the greater surface area available for enzymatic digestion. Over the past four decades, numerous clinical studies have investigated whether botanical structure preservation and reduced milling intensity improve postprandial glycemic and hormonal responses in minimally processed foods and reformulated products, including bread, biscuits, and pasta [99].

The clinical studies included in this review differ in whether particle size or cellular integrity could be independently examined independently from other compositional and processing-related characteristics. Studies in which structural characteristics were changed while other characteristics remained relatively similar provide more direct evidence of the effects of particle size or cellular integrity. In contrast, comparisons involving different botanical sources, formulations, or processing conditions reflect the combined effects of structural and compositional differences. Therefore, the findings are interpreted in relation to the overall food structure and processing conditions rather than being solely attributed solely to particle size or cellular integrity.

Figure 3 and Figure 4 summarize how the degree of milling and cell wall integrity in cereals and legumes influence particle size, starch encapsulation, the postprandial metabolic responses.

### 7.1. Cereals

The effect of cereal particle size on postprandial glycemic and other metabolic responses. Overall, most studies support a slowing effect of larger particle size or preserved grain structure, although the findings are not entirely consistent.

In wheat-based porridges, consumption of coarsely cracked whole wheat (≥1680 μm) has been associated with significantly lower postprandial glycemia than finely milled products (<150 μm), both in raw and gelatinized forms, in individuals with T2DM, highlighting the combined importance of structural preservation and limited thermal disintegration [100]. Similar findings have been reported in healthy volunteers, where porridge prepared from coarse wheat particles (2 mm) reduced postprandial glucose, insulin, C-peptide, and GIP responses compared with finely milled wheat flour (<200 μm). However, ileal recovery data indicated complete digestion of entrapped starch within 22 h, suggesting that particle size primarily slows the rate of starch digestion, rather than the final extent. GLP-1, PYY, cholecystokinin, and triglycerides were unaffected [101]. Consistent with these observations, fully gelatinized porridges prepared from cracked wheat (180–1700 μm) or semolina (425 μm) produced significantly lower glucose 120 min incremental area under the curve (iAUC) values than porridges prepared from fine flour, independent of viscosity differences, further supporting an independent role of particle size and grain refinement in glycemic regulation [102].

Comparable effects of particle size and structural integrity on postprandial metabolism have been observed across other cereal grains, particularly oats and rye. Consumption of porridge prepared from oat flakes significantly delayed gastric emptying and reduced postprandial plasma glucose, insulin, and GIP concentrations compared with porridge made from finely milled oat flour, although GLP-1 responses were unchanged [103]. Similarly, minimally processed steel-cut oats improved postprandial glycemic and insulinemic responses relative to instant oats, which undergo more intensive processing [104]. More recently, porridges prepared from coarse or whole oat fractions elicited significantly lower glycemic and insulinemic iAUC values and greater suppression of appetite sensations than fine oat flour porridges, further supporting the metabolic relevance of particle size and preserved cellular structure [105]. Consistent with these findings, the preservation of grain structure in rice has been associated with favorable glycemic effects. Brown rice produced significantly lower glycemic iAUC values than milled rice in both healthy individuals and those with T2DM, with reductions of approximately 19.8% and 35.2%, respectively [106].

However, the available evidence is not entirely consistent. In one clinical study involving healthy women, no significant differences in postprandial glucose or insulin responses were observed between porridges prepared from oat or barley flour and those made from their flaked counterparts. Nevertheless, botanical origin appeared to play an important role, as barley products (both flour and flakes) elicited significantly lower postprandial glucose and insulin responses than oat products, an effect attributed primarily to their higher soluble fiber content [107].

### 7.2. Legumes

Whole legumes are typically slow-digesting foods that retain much of their cellular structure during digestion. As a proportion of their starch remains entrapped within intact cell walls as RS1, they may exert favorable postprandial metabolic effects. Numerous in vivo studies have examined legumes in different physical forms to determine how structural preservation and processing influence glycemic, hormonal, and satiety responses [108].

Ileal recovery studies further demonstrate the physiological relevance of preserved legume structure. Following consumption of whole white beans, approximately 16.5% of ingested starch escaped small-intestinal digestion and reached the colon [109]. Lentils also contain RS, with their content varying according to lentil type and processing conditions [110]. These findings suggest that industrial processing conditions are critical in determining whether legume-based foods retain the metabolic benefits associated with whole seeds.

Several clinical studies have indicated that processing intensity strongly influences postprandial responses, largely through its effects on cellular integrity. Whole boiled lentils produced significantly lower glycemic responses than boiled, pureed, and dried, highlighting the metabolic importance of preserving native structure [111]. In contrast, another early study reported that ground and cooked lentils elicited a delayed glucose peak and lower overall glycemic response than whole lentils, despite similar insulinemic responses, illustrating that processing effects are not always uniform and may depend on preparation method and matrix characteristics [112]. The glycemic advantage of lentils was attenuated when they underwent more intensive processing, such as spray-drying followed by milling, consistent with the disruption of cellular barriers that normally limit starch accessibility [113]., Milling whole peas into flour resulted in significantly higher postprandial glycemic and insulinemic responses over 180 min, irrespective of genotype, further supporting the adverse metabolic impact of fine particle size [114].

Conversely, the processing strategies designed to preserve cellular architecture are more favorable. Red kidney beans that were boiled, freeze-dried, and subsequently milled induced lower glycemic responses than beans milled before steaming and freeze-drying, an effect attributed to more effective starch encapsulation within intact cell walls under the former process sequence [115]. Similar findings have been reported in individuals with T2DM, where minimally processed white beans with preserved cellular integrity reduced postprandial glucose and insulin concentrations, both when consumed alone and as part of mixed meals. Even in healthy individuals, unprocessed beans lowered insulinemic responses without significantly altering glycemia, suggesting potential benefits for everyday metabolic regulation [116], or recently, when whole legumes (lentils or peas) were compared with their flour equivalents in individuals with T2DM, all legume forms produced lower glycemic responses than a glucose reference drink, but early postprandial glycemia (0–60 min) was significantly lower after whole peas than pea flour [117].

However, not all studies have observed clear differences according to physical form. In one trial evaluating navy beans, chickpeas, and lentils as whole seeds, purees, or powders, no significant differences in postprandial glycemia were detected either acutely or after a standardized second meal consumed 2 h later [118]. Similarly, there were no significant differences in glycemic or hormonal responses (GLP-1, C-peptide, resistin), or in subjective appetite ratings between whole chickpeas, chickpea puree, and chickpea pasta. Nevertheless, whole chickpeas and puree reduced glycemic responses to the subsequent meal, possibly due to higher slowly digestible starch content and increased SCFA production associated with greater structural preservation [9]. These findings contrast with those of earlier studies that reported clearer processing-related differences [111,112,115].

More recent studies using novel chickpea cell powders have provided evidence for the importance of cellular integrity. Beverages prepared from chickpea powder containing intact cells produced significantly lower 30-min glucose peaks than beverages made from powders with disrupted cells, despite similar glucose iAUC_120_ values [7,50]. A similar pattern was observed following the consumption of chickpea puree prepared from flour containing intact cells, which significantly reduced hunger, desire to eat, and prospective food consumption, while increasing fullness and satiety relative to conventional flour [10]. In addition, hummus prepared from chickpeas with preserved cellular structure elicited significantly lower postprandial insulin and GIP responses than hummus made from disrupted cells, highlighting the role of cellular integrity in regulating appetite-related metabolic and hormonal responses [74].

These observations are further supported by evidence from double-blind randomized crossover trial in which isoenergetic chickpea meals differed only in microstructure (i.e., intact cellular aggregates, separated cells, or disrupted cells). Meals containing disrupted cells elicited substantially greater and more prolonged insulinemic responses, with approximately 65% higher peak insulin concentrations and 74% higher insulin iAUC than meals containing intact cellular aggregates. The microstructure also altered incretin kinetics: disrupted-cell meals produced a rapid early GIP response, whereas intact-cell meals generated a slower, more sustained pattern with lower peak and total GIP exposure. Intact-cell meals delayed GLP-1 responses and increased overall GLP-1 exposure despite similar peak concentrations. In healthy participants, cellular structure preservation was also associated with lower desire to eat and reduced overall appetite scores, although subsequent energy intake was unchanged [119].

### 7.3. Starchy Foods: Bread, Bakery Products, and Pasta

A substantial number of in vivo studies have examined whether replacing part of conventional finely milled flour (<200 μm) with coarser cereal fractions or structurally preserved grain ingredients can modify postprandial metabolic responses in healthy individuals and those with T2DM. Since the 1980s, growing evidence has indicated that glycemic responses are determined not only by nutrient composition but also by the extent to which botanical and cellular structure is retained during processing.

Jenkins et al. demonstrated that incorporating 80% of whole wheat kernels into pumpernickel bread significantly lowered the GI compared with bread prepared solely from finely milled wholemeal flour [120]. In a subsequent study, the inclusion of whole or cracked wheat and barley kernels similarly reduced GI, with greater effects observed at higher substitution levels (75% for wheat and 50% for barley) [121]. Barley-enriched bread consistently produced lower glycemic responses, likely reflecting their higher content of soluble fiber, particularly β-glucan. More recently, Reynolds et al. (2020) reported that bread containing 50% coarsely cracked whole grains (1265 μm) elicited lower GI and glycemic iAUC values in individuals with T2DM, with an even stronger effect when the formulation included both cracked wheat (30%) and intact wheat kernels (30%; 2240 μm) [15].

In contrast, studies in metabolically healthy individuals have produced more heterogeneous findings. In adults with sedentary lifestyles, overweight/obesity, and T2DM risk factors, no significant differences in postprandial glucose or insulin iAUC_180_ were observed between breads prepared from coarse versus fine stone-milled flour [122]. Similarly, wholegrain bread containing 50% flour fractions of 150–850 μm did not differ from ultrafine wholegrain or white bread in postprandial glucose, insulin, or glucagon responses over 3 h [123], and no differences were detected among breads prepared from fine, medium, or coarse wheat flour [124]. In a recent study, wheat biscuits made with either conventional fine flour or coarse flour (>1000 μm) showed no differences in glucose iAUC_180_ or peak glucose concentrations, despite the coarse product containing approximately twice as much SDS. However, significant differences at 90 and 120 min indicated a redistribution of glycemic response over time rather than a reduction in total exposure [36]. This inconsistency may partly reflect tighter insulin and incretin regulation in healthy individuals, which can mask structural effects on circulating glucose.

Nevertheless, significant benefits have also been reported in healthy cohorts, who had lower glycemic and insulinemic indices after consumption of breads containing 80% preboiled whole kernels of wheat, rye, or barley plus 20% white wheat flour [125]. When white flour was replaced with oats at the same level, glucose iAUC_120_ was unchanged, but insulin responses were reduced, suggesting that food structure may influence insulinemia even when glycemia remains stable. O’Dea et al. found that both healthy ileostomy participants and individuals with T2DM exhibited lower postprandial glucose and insulin responses after coarse wheat bread compared with finely milled bread. Higher levels of undigested starch recovered from ileal effluent supported partial preservation of starch encapsulation and slower digestion [112].

Similarly, muffins formulated with intermediate-particle-size sorghum flour exhibited significantly lower GI than those prepared with finely milled sorghum flour or comparator grains such as rice, likely reflecting particle-size-dependent structural differences formed during baking [126].

The food matrix itself also appears to be critical. The incorporation of a 60% mixture of cracked and intact wheat kernels (1:1) into crackers reduced postprandial glucose by 36% compared with crackers made solely from roller-milled flour. The same formulation in bread had no significant effect, nor did the porridge, which was entirely prepared from cracked wheat in the same protocol [127]. The favorable response in crackers was attributed to their lower moisture content, which likely limited starch hydration and gelatinization during baking. Consistent with this observation, enrichment of wheat bread with coarse bran significantly lowered GI (69.0 ± 8.1) compared with conventional wheat bread (85.0 ± 6.6) and bread containing an equivalent amount of fine bran (82.0 ± 9.9), highlighting the relevance of bran particle size [128].

Comparable findings have also been reported for rye products. Whole-kernel rye bread induced lower postprandial glucose and insulin responses than white wheat bread, although no significant effects were observed on postprandial ghrelin concentrations, subjective appetite ratings, or subsequent energy intake at an ad libitum lunch meal. Increased hydrogen excretion during the late postprandial phase (270–390 min) suggested enhanced colonic fermentation, consistent with the delivery of undigested carbohydrates to the large intestine [129].

Beyond glycemia, cereal particle size may also influence insulin secretion, incretin responses, and appetite regulation. Scones prepared from whole or cracked cereal grains, or from coarse flour (wheat, maize, oat), produced similar glucose responses but significantly lower insulin concentrations, with oat scones showing the lowest values, possibly due to greater preservation of grain microstructure during baking [81]. In contrast, compared with white and wholemeal breads, bread prepared from preboiled whole wheat kernels increased subjective appetite ratings despite similar glycemic responses and gastric emptying [38]. A more recent study found no significant differences in glucose iAUC or satiety ratings between coarse- and fine-flour breads, although coarse breads tended to produce lower GI values (55–69 vs. 70–102 for white bread) [130]. Whole-kernel formulations in rye breads did not alter glycemia or gastric emptying, but reduced postprandial insulin, GLP-1, and GIP responses compared with wholemeal or white wheat breads [131]. Similarly, breads in which 85% of wheat flour was replaced with cracked kernels elicited comparable glucose, insulin, and GIP kinetics over 360 min, but significantly lower GLP-1 iAUC values [8].

Clinical evidence involving coarse legume ingredients in baked products remains limited but promising. In one study, standard wheat bread enriched with 37.5% soy flour and 37.5% coarse wheat particles (>2.8 mm) did not alter glycemic responses when consumed under normal mastication. However, when the bread was swallowed without chewing, GI was significantly lower, consistent with greater preservation of structural integrity and suggesting that oral breakdown may partly negate the metabolic advantage conferred by coarse particles [77]. In another study, substituting 30% of either refined wheat flour or conventional chickpea flour (<200 μm) with large-particle-size chickpea flour (1400–1800 μm) significantly reduced postprandial glycemic responses, increased GLP-1 iAUC, and enhanced subjective appetite ratings compared with control white bread. In contrast, the replacement of refined wheat flour with cracked whole wheat did not significantly affect these outcomes, suggesting that the preservation of legume cellular structure confers greater metabolic benefits than coarse cereal particles alone [132].

Further studies showed that replacing 30% or 60% of wheat flour with chickpea flour containing intact cells reduced postprandial glycemic responses by approximately 40%, with no clear difference between substitution levels, indicating that 30% enrichment may be sufficient to achieve metabolic benefit [7]. In a subsequent trial, only the 30% substitution significantly reduced glucose iAUC, whereas the 60% chickpea bread elicited lower insulin and C-peptide iAUC values together with higher GLP-1 and PYY responses. Enriched breads also produced higher postprandial concentrations of total and essential amino acids, indicating that a significant proportion of protein remained bioaccessible despite partial cellular encapsulation [97,98]. Table 4 provides an overview of the intervention studies that assessed the influence of particle size and cell integrity on the regulation of postprandial glucose.

### 7.4. Long-Term Interventions

To date, few long-term intervention studies have examined whether cereal processing and grain structure influence postprandial metabolic responses and body weight regulation beyond acute postprandial studies. In individuals with T2DM, a 24-day comparison of low- versus high-GI diets that differed primarily in the degree of milling of carbohydrate-rich foods showed a more favorable metabolic profile with the low-GI, less processed diet, including lower postprandial glucose, insulin, and C-peptide concentrations, reduced LDL-cholesterol, and improved fibrinolytic activity [134].

Similarly, a 6-week trial comparing whole-grain and refined-grain diets reported higher resting metabolic rate, greater fecal energy losses, a modest improvement in glycemia following an oral glucose tolerance test, and progressive reductions in prospective food consumption in the whole-grain group [135]. However, interpretation is limited because commercially available whole-grain products with insufficiently characterized structural properties (e.g., flour particle size, and kernel integrity) were used, making it difficult to distinguish the effects of grain structure from those of higher dietary fiber content.

A 2-week crossover study in adults with T2DM compared whole-grain foods with similar nutrient composition but differing processing degree. The less processed cereal diet, including oats, wheat breads, and rice, resulted in lower postprandial glycemia, particularly after breakfast, reduced glycemic variability, and modest weight loss despite similar energy intake [14]. These findings highlight the possibility that intact or minimally processed grains may partially escape small-intestinal digestion, increase colonic fermentation, or require greater digestive effort, thereby influencing energy balance and glycemic control.

Current available evidence indicates that reducing cereal processing may confer benefits for metabolic regulation and body-weight management. However, well-controlled long-term trials are needed to isolate the independent contribution of particle size and structural integrity from accompanying differences in fiber content, palatability, and food composition.

### 7.5. Systematic Reviews and Meta Analyses

A limited number of systematic reviews and meta-analyses have examined the impact of cereal grain structure and processing on postprandial metabolic responses, highlighting the importance of particle size, matrix integrity, and degree of processing in modulating glycemic outcomes.

A systematic review examining oat processing in terms of milling and cooking, found, that minimally processed oats, such as large flakes, steel-cut oats, and muesli/granola, exhibited substantially lower GI values (55, 56, and 53, respectively) compared with instant and quick oats (GI 71 and 75, respectively) [136]. These differences were attributed to the smaller particle size, greater disruption of oat grain structure, and increased starch gelatinization during cooking. A subsequent systematic review and meta-analysis of randomized controlled trials similarly reported that intact oat kernels and thick oat flakes significantly reduced postprandial glycemic and insulinemic responses compared with refined-grain controls, whereas thin, quick, or instant oat flakes conferred no significant benefit, suggesting that the metabolic advantages of oats are attenuated by progressive disruption of oat kernel structure [137].

A recent meta-analysis also demonstrated that starchy foods prepared with larger-particle flours, resulting from lower industrial milling intensity or reduced oral breakdown, significantly attenuated postprandial glucose and insulin responses. The effects on subjective appetite ratings and gut hormone secretion were more variable, although a consistent reduction in GIP concentrations was observed [16,133]. Comparable findings were reported in a systematic review and meta-analysis comparing whole-grain with refined wheat, rice, and rye products. Consumption of intact whole-grain rice was associated with significantly attenuated postprandial blood glucose responses, whereas wholemeal wheat and rye products showed no consistent benefit over refined counterparts. This likely reflects the fact that wheat and rye were predominantly consumed as milled flour rather than as intact kernels, highlighting the importance of preserving grain structure rather than relying solely on whole-grain composition [138].

Although acute consumption of whole-grain foods has been frequently associated with improved glycemic and insulinemic responses, long-term interventions have not consistently demonstrated similar benefits. Many analyses classified foods according to the presence of bran, germ, and endosperm without accounting for structural integrity (e.g., particle size, intact cell walls, or degree of processing), which may have limited the ability to detect physiologically relevant processing-related differences.

## 8. Discussion

Although dietary recommendations and food classification systems largely rely on nutrient content, evidence indicates that the structural organization of plant foods also influences nutrient accessibility, starch digestibility, and postprandial metabolic responses. Preserving cellular integrity is an important determinant of starch bioaccessibility and digestibility across cereals, legumes, and other starch-rich foods, although its effects on postprandial responses may vary with food composition, processing, and physiological conditions. The available findings highlight the importance of considering the physical organization of nutrients within the food matrix when evaluating the nutritional functionality of carbohydrate-rich foods. In particular, starch digestibility and postprandial metabolic responses cannot be fully explained by composition alone. In cereal- and legume-based foods, cellular structure changes induced by processing can strongly influence nutrient accessibility and, consequently, physiological responses. This concept has important implications for current whole-grain classifications, which are based primarily on the presence and relative proportions of bran, germ, and endosperm, irrespective of the degree of structural preservation. Foods meeting the same compositional criteria may differ in their digestive and metabolic behavior. The available evidence discussed in this review suggests that consideration of food structure, alongside composition, may provide a more physiologically relevant framework for evaluating the nutritional functionality of cereal- and legume-based foods and distinguishing products with similar composition but different metabolic outcomes [15].

Plant cell walls provide an important physical barrier to digestion by limiting hydration, swelling, and enzyme access to intracellular starch [30]. Mechanical processing, particularly fine milling, increases the surface area, ruptures cells, and accelerates starch hydrolysis. Roller milling, the dominant industrial technology, can cause substantial microstructural damage, and even moderate reductions in particle size may increase in vitro starch digestibility. However, a central conclusion emerging from the available evidence is that particle size should not be regarded as a direct proxy for structural integrity. Materials with similar particle-size distributions may differ markedly in the proportion of intact cells, starch encapsulation, damaged starch content, hydration behavior, gelatinization characteristics, and resistance to oral processing [77].

Subsequent processing steps can significantly modify the structural effects created during milling. Baking, steaming, extrusion, flaking, and other hydrothermal treatments promote starch gelatinization, alter water distribution, increase cell wall permeability, and facilitate enzyme diffusion through disrupted matrices. Therefore, digestibility should be viewed as the consequence of cumulative structural changes occurring throughout the production chain rather than milling intensity alone. This helps explain why foods produced from similarly milled ingredients may differ in terms of glycemic impact. These observations also have implications for current discussions on food processing, as different processing operations can have distinct effects on food structure but are i often considered as a single characteristic. The evidence reviewed here suggests that even relatively simple processes, such as milling can influence nutrient accessibility and metabolic responses through their effects on plant microstructure. Therefore, consideration of structural disruption alongside conventional compositional and formulation-based criteria may provide a more physiologically relevant framework for evaluating the health effects of food processing.

Oral processing represents an additional stage at which food structure can be modified before enzymatic digestion occurs. Although cellular integrity is largely preserved during manufacturing, mastication and gastric mixing may disrupt cellular barriers and increase starch accessibility. Conversely, structurally resilient particles can retain encapsulated starch throughout the upper gastrointestinal tract, allowing a proportion to reach the colon for fermentation. These observations highlight that the metabolic effects of food structure depend not only on the degree of processing but also on the persistence of cellular integrity during oral and gastric disintegration [26,69].

Taken together, postprandial responses to cereal- and legume-based foods arise from the combined effects of industrial processing, domestic preparation, oral processing, and gastrointestinal digestion. Particle size should not be considered an isolated causal factor, but rather an indicator of broader structural integrity. Foods with similar mean particle size may differ substantially in particle size distribution, ruptured cells proportion, starch gelatinization, hydration behavior, and mastication resistance, all of which may influence glycemic and hormonal responses.

Botanical origin is another critical determinant of carbohydrate functionality. Differences in cell wall composition, thickness, porosity, and permeability between cereals and legumes affect resistance to mechanical and hydrothermal disruption [72]. Legume cotyledon cell walls are typically thicker and richer in pectic polysaccharides than cereal endosperm cell walls, conferring greater structural resilience during processing and digestion. Consequently, legume flours frequently exhibit lower starch digestibility than cereal flours with comparable particle size [29]. From a formulation perspective, legumes may retain functional benefits under processing conditions that diminish structural effects in cereals. These intrinsic structural differences highlight the importance of considering botanical sources alongside particle size when designing starchy foods with improved metabolic functionality.

Despite these advantages, the incorporation of structurally intact or coarsely milled ingredients presents important technological and sensory challenges. In bakery systems, coarse particles often impair dough rheology, reduce loaf volume, and produce denser or firmer crumb structures [20,139]. These effects have been commonly attributed to competition for water, interruption of gluten network continuity, and reduced gas retention during proofing and baking [41,65]. Such changes may also modify oral processing behavior by increasing chewing effort and particle breakdown, thereby partly offsetting the intended metabolic benefits. These constraints remain a key barrier to consumer acceptance and large-scale industrial implementation. Accordingly, sensory evaluation using trained panels and consumer testing is essential for determining the acceptability of structure-preserving formulations and identifying whether consumers are willing to adopt such products under realistic eating conditions.

Although in vitro studies have consistently shown that larger particle size and preserved cellular integrity reduce starch digestibility, translation to in vivo outcomes has been less consistent. Some studies have reported little or no reduction in postprandial glycemia after the incorporation of coarse flour into bread, even when in vitro digestion is slower. This discrepancy may reflect the fact that the insufficiency of particle size in predicting physiological response [13,65]. Mixing, fermentation, and baking may weaken cell walls and gelatinize starch sufficiently to diminish the protective effect of coarse particles. In addition, inclusion levels may be too low to significantly alter the structural properties of the final product, or compensatory mastication may increase particle disruption before digestion. In contrast, more consistent benefits are often observed when intact kernels or larger structurally resilient particles are incorporated, particularly in individuals with impaired glucose regulation, such as those with T2DM [14,15,77,100,121].

Conflicting findings regarding the functional properties of legume flours, including WHC, viscosity, and digestibility, may similarly reflect competing mechanisms. Particle size reduction can increase hydration rate, swelling, and viscosity, which may slow digestion, while simultaneously increasing surface area and enzyme accessibility, which may accelerate digestion. Therefore, the net effect is unlikely to be linear and depends on the botanical source, protein–starch interactions, fiber structure, and the extent of cellular rupture during milling.

A major limitation of the current literature is the predominance of acute studies conducted in young and metabolically healthy participants, in whom subtle structural effects may be masked by high insulin sensitivity and efficient mastication. Long-term interventions remain scarce and are often too short to detect meaningful changes in body weight, insulin sensitivity, appetite regulation, or cardiometabolic risk. Additional heterogeneity arises from inconsistent reporting of particle size distribution, inadequate characterization of structural integrity, differences in prior hydrothermal treatment, and inter-individual variation in oral processing behavior. Moreover, relatively few studies have assessed postprandial insulin responses, incretin hormones, appetite, or subsequent energy intake alongside glycemia, despite evidence that reductions in insulinemia may confer metabolic benefits even when glucose responses are unchanged [8,131,140]. In vivo evidence for coarsely milled legume-based foods remains limited, despite their higher structural integrity and potential to enhance satiety and metabolic outcomes [7,77,132].

From a clinical perspective, these findings suggest that food structure may be considered as an additional factor when selecting or developing carbohydrate-rich foods for dietary management, particularly when postprandial glycemic control is an important consideration. Foods with preserved cellular integrity and larger structural units may offer a potential approach for moderating starch digestion and postprandial metabolic responses. However, the available human evidence remains heterogeneous, and the potential clinical relevance of these structural characteristics requires further investigation. Therefore, structural characteristics should be regarded as a complementary aspect of dietary management rather than as a stand-alone strategy. These findings also suggest that structural characteristics may serve as additional design parameters in the development and reformulation of cereal- and legume-based foods, provided that nutritional effects are evaluated together with technological and sensory performance.

Future research should prioritize both acute and long-term clinical trials using structurally characterized cereal- and legume-based foods, particularly legume-enriched staple products, as practical strategies to increase legume intake and reduce the glycemic impact of commonly consumed high-GI foods. Studies should better reflect real-world eating patterns by testing foods within mixed meals, assessing second-meal effects, and incorporating measurement of insulin, GLP-1, GIP, PYY, rate of gastric emptying, fermentation, and gut microbiome responses. Equally important is the optimization of processing technologies to identify formulations containing sufficient proportions of structurally intact particles that remain functional after manufacture and mastication while maintaining acceptable sensory quality. Addressing these issues is essential for translating structure-based concepts into practical dietary strategies.

## 9. Conclusions

Preservation of native plant structure through reduced milling intensity represents a promising strategy for improving the metabolic quality of starchy cereal- and legume-based starchy foods. In vitro evidence indicates that retention of intact or partially intact cellular structures can increase the proportion of slowly digestible and physically inaccessible starch, thereby slowing starch hydrolysis. However, human evidence is less consistent, indicating that changes in starch digestibility do not necessarily translate directly into lower postprandial glycemic responses. These effects have been observed across a range of foods, including breads, porridges, pasta, and cereal–legume products, although their magnitude depends on the population’s physiological characteristics, botanical origin, formulation, and processing conditions.

From a practical perspective, these findings support greater consideration of food structure alongside nutrient composition in dietary guidance and food reformulation. However, cellular structure preservation should not be viewed as a stand-alone strategy, and potential metabolic benefits must be balanced against technological and sensory constraints, as the excessive incorporation of coarse fractions may adversely affect product quality. Future food reformulation strategies could focus on optimizing particle size, cellular integrity, and processing conditions to improve starch functionality while maintaining consumer acceptability.

A better understanding of how structural characteristics interact with individual metabolic responses may also contribute to precision nutrition approaches, although current human evidence remains insufficient to define population-specific recommendations. Similarly, grain structure and milling characteristics may be relevant for breeding and selection of cereal and legume varieties with desirable nutritional and technological properties. Overall, the composition of carbohydrate-rich foods and the way their native structure is modified throughout industrial processing may influence their nutritional functionality.

## Figures and Tables

**Figure 1:**
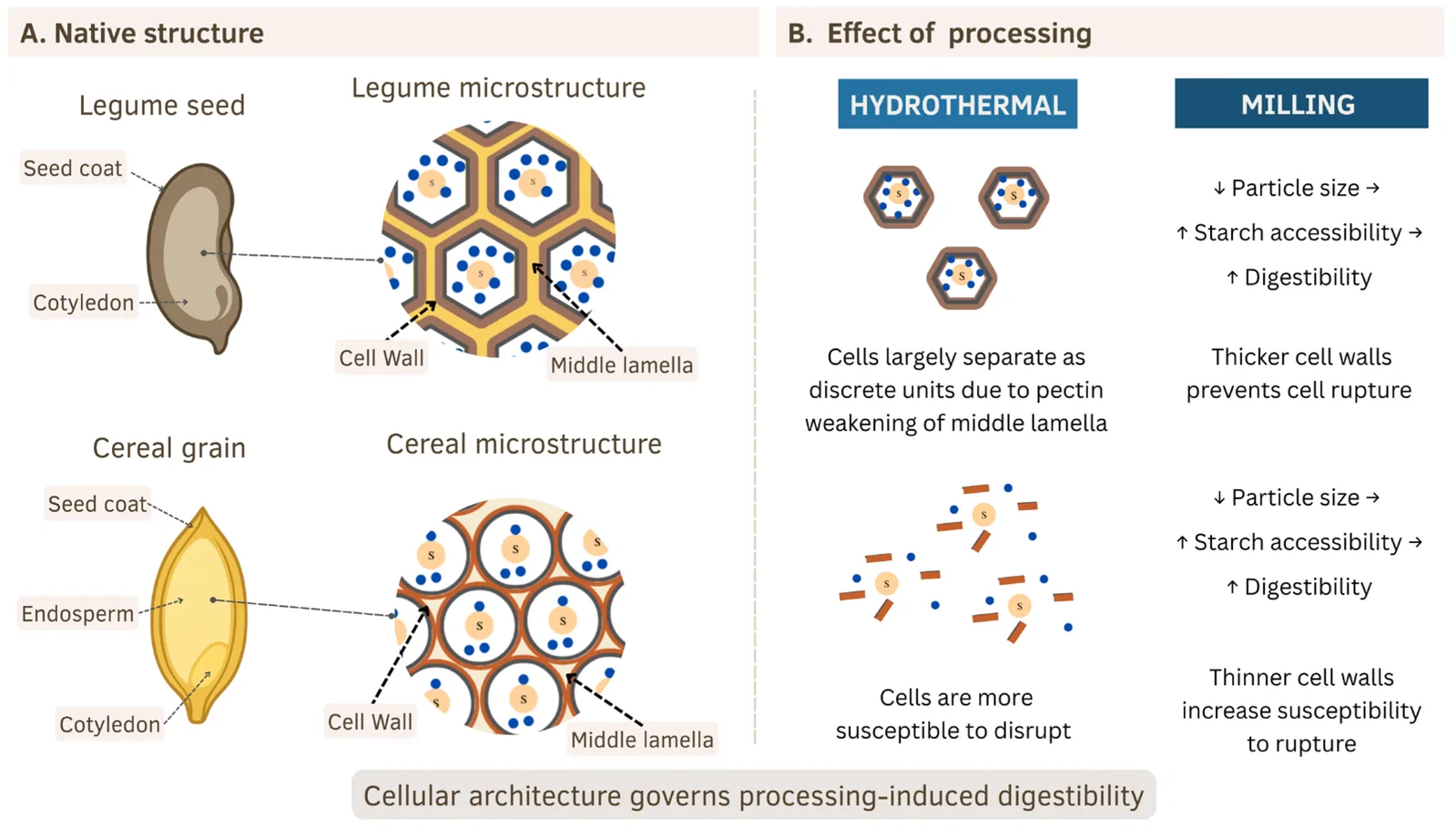
Summary of the effects of food structure (particle size, cellular integrity, and processing) on starch digestibility and structural changes during digestion. Findings are grouped based on the sample type and processing conditions. Starch granules are shown as orange circles marked with the letter “S”, while proteins are shown as blue circles.

**Figure 2:**
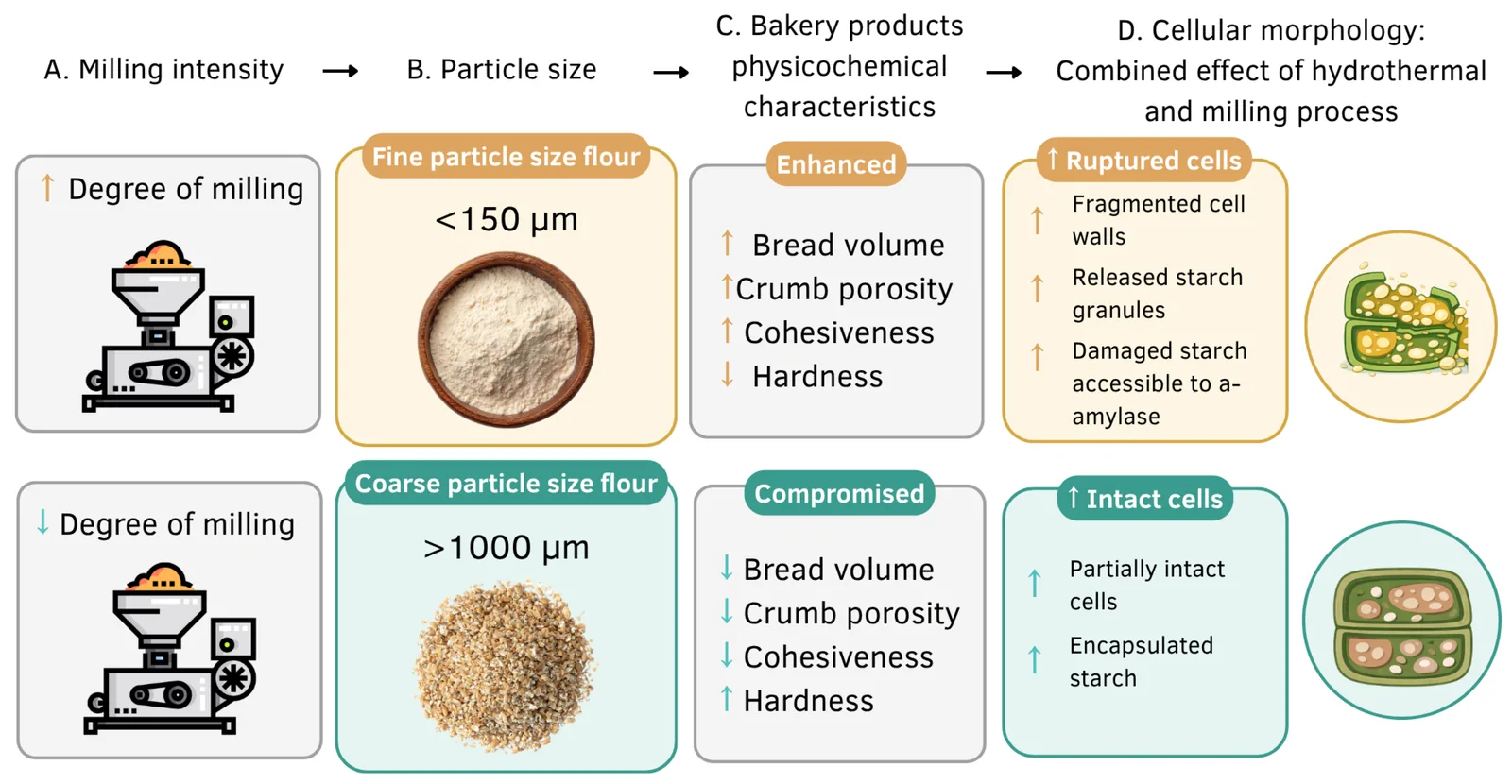
Schematic representation of the effect of milling intensity on the particle size, physicochemical properties, and cellular morphology. Increased milling intensity leads to finer particles (<150 μm), resulting in enhanced bread quality attributes (volume, porosity, cohesiveness) but increased cell wall disruption and starch accessibility. In contrast, coarser particles (>1000 μm) retain more intact cellular structures, limiting starch accessibility and modifying product texture.

**Figure 3:**
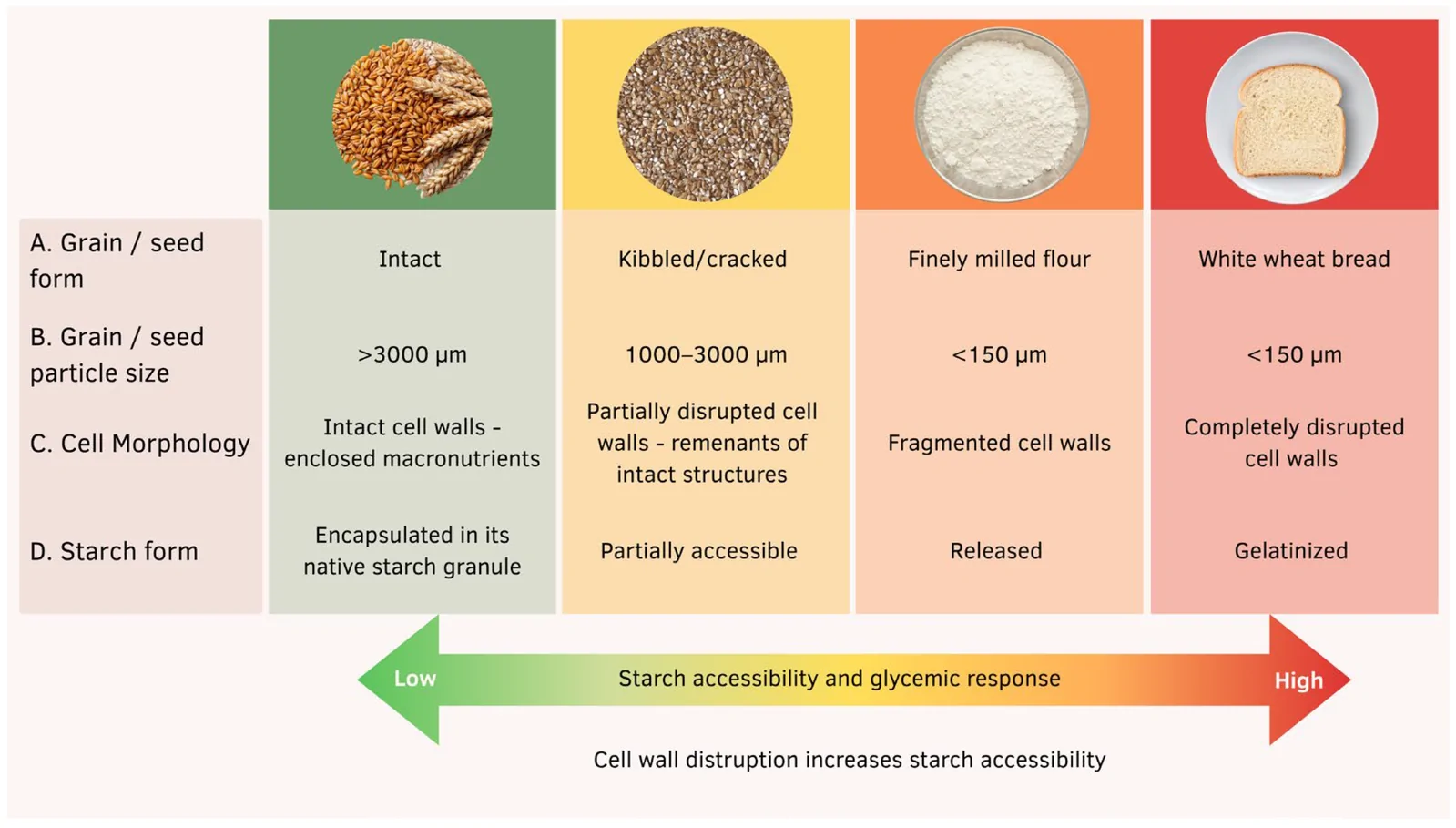
Structural continuum from intact grains to refined bakery products illustrating the impact of particle size reduction and cell wall disruption on starch accessibility. Intact grains (>3000 μm) maintain cellular encapsulation of starch, whereas milling and processing progressively disrupt cell walls, resulting in partially accessible, released, and ultimately gelatinized starch. This transition is associated with increased starch digestibility and glycemic response.

**Figure 4:**
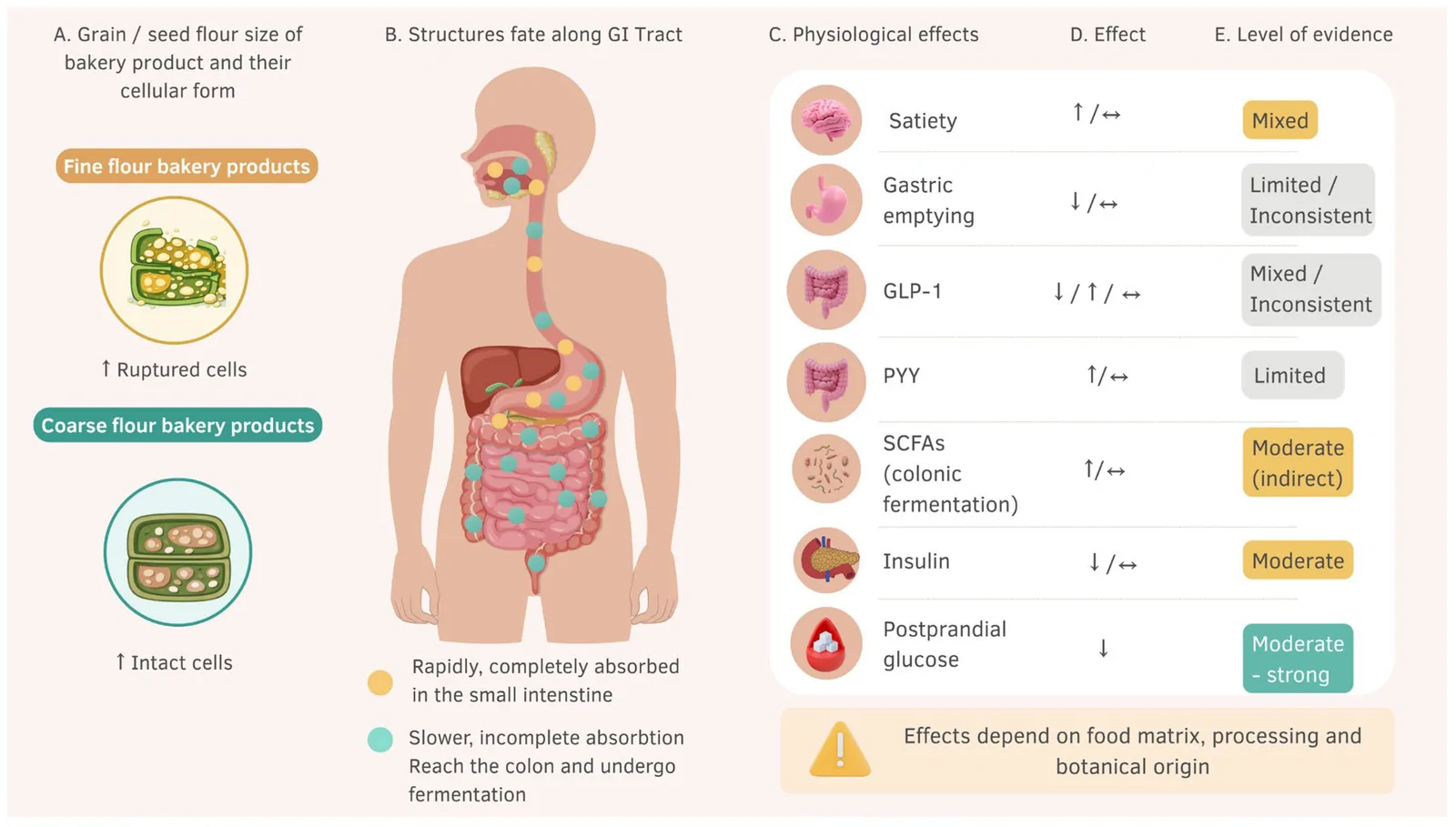
A conceptual overview of the gastrointestinal fate of cereal and legume structures with different degrees of cellular integrity and their associated physiological effects is presented. Fine particles and disrupted cells are generally more rapidly digested and absorbed in the small intestine, whereas intact cellular structures from coarse materials are more resistant to digestion and can reach the colon, where they may undergo fermentation. These pathways reflect mechanisms described in the existing literature, whereas the magnitude and occurrence of these effects depend on the food matrix, processing conditions, botanical origin, and individual physiological factors. Importantly, the figure distinguishes proposed mechanistic pathways from physiological effects demonstrated in human studies. The presence of intact cellular structures does not imply that they routinely reach the colon. Reported physiological outcomes (e.g., satiety, glycemic response, and SCFA production) vary in consistency depending on the food matrix, processing conditions, and study design.

**Table 1:** Comparative structural and compositional characteristics of cereals and legumes and their relevance to functionality, starch digestibility, and postprandial metabolic responses.

Characteristic	Cereals	Legumes
Principal storage tissue	Starchy endosperm [17,18]	Cotyledons [19,22]
Botanical classification	Monocotyledonous seeds [18]	Dicotyledonous seeds [19]
Cell size	Generally smaller Approximately 80 μm reported for wheat endosperm [11,17,18]	Generally larger Approximately 100–200 μm reported for legume cotyledons [19,22]
Major anatomical fractions	Bran, endosperm, germ [18,19]	Seed coat (testa), cotyledons, and embryonic axis
Kernel/seed morphology	Elongated or oval, often with a longitudinal crease [18]	Irregular and thicker structure (particularly chickpeas) [19]
Cell wall type	Type II primary cell wall [24]	Type I primary cell wall [24,25]
Cell wall polysaccharide profile	Rich in arabinoxylans and β-glucans Relatively low in pectin [17,24]	Rich in pectin and xyloglucans Lower-to-moderate lignin content [19,24]
Cell wall thickness	Variable among species and anatomical regions Generally thinner in endosperm cells [17,24]	Variable among species and tissues Generally thicker in cotyledon cells [24,25]
Cell wall porosity	Generally higher [24,30]	Generally lower [27,30]
Predominant storage proteins	Prolamins and glutelins [17,18]	Albumins and globulins [19,22]
Relative protein content	Generally lower in starchy endosperm; higher in bran, aleurone, and germ fractions [17,18]	Generally higher in cotyledons; substantial variation among species [19,22]
Relative lipid content	Generally lower in starchy endosperm Mainly concentrated in germ and aleurone [18]	Generally higher than in the cereal endosperm [22]
Predominant dietary fiber profile	Mainly insoluble fiber particularly in bran [17,18]	Soluble and insoluble fiber [19,31]
Starch digestibility	Generally higher	Generally lower
First-limiting amino acid	Lysine [18]	Sulfur-containing amino acids (e.g., methionine, and cysteine) [18,19]
Starch granule organization	Starch granules embedded in a protein matrix	Densely packed starch granules entrapped within the protein and fiber matrices
Response to hydrothermal processing	Greater cell rupture and starch release	Greater cell separation with partial cellular integrity retention

Note: The characteristics presented in this table represent general structural and compositional tendencies rather than universal differences between cereals and legumes. Considerable variation exists among species, cultivars, anatomical fractions, and processing conditions. The reported ranges should be considered representative rather than definitive.

**Table 2:** Effects of different milling methods on cellular integrity, starch accessibility, and digestibility and related properties.

Characteristic	Stone Milling	Roller Milling	Wet Milling	Cell Powder/Preserved Structure
Processing principle	Grinding between the millstones	Grinding between counter-rotating rollers, followed by sieving and classification	Processing under high-moisture conditions, often involving fractionation	Hydrothermal and/or wet fractionation processes
Particle size distribution	Relatively narrow distribution	More controlled particle-size reduction and fractionation	Variable Dependence on moisture and fractionation conditions	Fine particles containing intact plant cell aggregates
Cellular wall disruption	Maybe partially preserved, depending on milling intensity and moisture conditions	Variable to extensive depending on the milling intensity and roller settings	Potentially limited under hydrated conditions	Generally preserved to a greater extent
Starch granule damage	May remain partially restricted when cellular structures are preserved	May increase with greater tissue disruption and starch damage	May remain restricted when cellular structures are preserved	Generally restricted when cellular encapsulation is maintained
Potential effect on starch digestibility	May be lower when cellular integrity is preserved	May be increased by extensive structural disruption and starch damage	Strong dependence on processing conditions	Reduced when plant cell walls remain intact
Main factors influencing the outcome	Milling intensity, moisture content, temperature, mill design, and sieving	Roller configuration, pressure/gap, moisture, temperature, and sieving	Moisture, temperature, mechanical energy and fractionation	Cellular integrity and processing intensity

Note: The reported structural and digestive consequences represent general tendencies and potential effects rather than inherent properties of each milling approach. The actual outcomes depend on the botanical material and processing conditions, including moisture, temperature, mechanical energy, mill configuration, and fractionation procedures.

**Table 4:** Human studies examining postprandial glucose responses to cereal- and legume-based products enriched with larger particles or intact cells.

Cereal/Legume Type	Particle Size/Structure	Test Product	Reference Food	Available CHO ^a^	Subjects	Glycemic Response	Other Metabolic Responses	Appetite Ratings	Ref.
Chickpea	Intact chickpea cells (ICC) Ruptured chickpea cells (RCC)	Hummus of (ICC) † (RCC) †	No external control	Not reported (matched with 27 g starch)	Healthy normal-weight subjects (n = 15)	↔ iAUC_180_	iAUC_180_ Insulin: ↓ ICC (* vs. RCC) GLP-1, GIP: ↔	Not measured	[74]
Wheat Chickpea	Chickpea intact cell powder (ICP) Chickpea intact cell powder (RCP)	Drink with ICP † Drink with RCP †	Glucose drink	58 g	Healthy subjects with a BMI of 18–35 kg/m^2^ (n = 20)	Interstitial glucose (CGM) (mmol/L × min) iAUC_45_: ↓ ICP vs. RCP (40%); ↓ ICP, RCP (* vs. glucose drink) iAUC_120_: ↓ ICP, RCP (* vs. glucose drink)	Not measured	Not measured	[7]
Wheat Chickpea	Chickpea intact cell powder (ICP), 30% or 60% replacement Wheat flour	30% ICP bread (ICP30) ‡ 60% ICP bread (ICP60) ‡	0% ICP bread (ICP0)	50 g	Healthy subjects with a BMI of 18–35 kg/m^2^ (n = 16)	Interstitial glucose (CGM) (mmol/L × min) iAUC_120_ min: ↓ ICP (30 or 60) (−40% * vs. ICP0); ICP30 ≃ ICP60 (NS)	Not measured	Not measured	[7]
Wheat Chickpea	Chickpea intact cell powder (ICP) 30% or 60% replacement	30% ICP bread (ICP30) ‡ 60% ICP bread (ICP60) ‡	0% ICP bread (ICP0)	50 g	Healthy subjects with a BMI of 18–35 kg/m^2^ (n = 20)	iAUC_120_ min: ↑ ICP0 (* vs. ICP30, ICP60) iAUC (1st peak): ↓ ICP30 (>40% * vs. ICP0; ICP60 (NS vs. ICP0)	iAUC_120_ min Insulin: ↓ ICP (* vs. ICP0, ICP30); ↑ GLP-1 (* vs. ICP0, ICP30) GIP: ↔ PYY: ↑ (* vs. ICP0, ICP30)	↔ Combined satiety score for all test breads	[97]
Wheat	White wheat flour Whole grain wheat flour Ultra-fine ground whole grain wheat flour	White wheat bread (WB) ‡ Whole wheat bread (WWB) † Ultra-fine whole wheat bread (UFWB) †	Glucose solution	Not reported (1 g CHO/kg BW)	Healthy, normal-weight subjects (n = 26)	AUC_120 min_: ↑ Glucose (* vs. WB, WWB, UFWB)	iAUC_180 min_: Insulin: ↔ for all test meals Glucagon: ↓ Glucose solution (* vs. all test breads); ↑ WB (* vs. WWB, UFWB); ↓ WWB, UFWB (* vs. WB)	Not measured	[123]
Oats Barley	Oat flakes Oat flour Barley flakes Barley flour	Porridges of oat flakes Oat flour Barley flakes Barley flour	Glucose solution	Not reported	Healthy overweight–obese subjects (n = 10)	AUC_120 min:_ ↑ Glucose (* vs. all test meals); ↑ Oat flakes porridge (* vs. barley flour and flakes) ‡ ↔ flakes vs. flour within oats and barley †	AUC_120 min_ Insulin: ↓ in barley flakes, flour porridges (* vs. glucose, oat porridges) ‡; ↔ flakes vs. flour; ↔ Glucagon, leptin for all test meals	Not measured	[107]
Chickpea	Intact cell clusters (ICC) Intact separated cells (ICS) Broken cells (BC)	Porridges of ICC † ICS † BC †	No external control	Not reported (30 g starch per serving)	Healthy normal-weight subjects (n = 15)	iAUC_180 min_: ↑ BC (+148%) * vs. ICC; ↔ ICC vs. BS	iAUC (ipeak) Insulin: ↑ BC (+74%) * vs. ICC GIP: ICC (−54%), ICS (−61%) * vs. BC GLP-1: ↑ ICS (+63%) * vs. BC PYY: ↑ ICC * vs. BC	iAUC_180 min_ Fullness: ↑ for ICS (* vs. ICC and BC) Food intake: ↔ for all test meals	[119]
Wheat	Coarse Fine	Porridge in coarse † and fine † forms	No external control	57.8 g	Healthy ileostomy participants (n = 9)	iAUC_120 min:_ ↓ coarse vs. smooth (−33%)	iAUC_120 min_ Insulin: ↓ coarse (−43% * vs. smooth) C-peptide: ↓ coarse (−40% * vs. smooth) GIP: ↓ coarse (−50% * vs. smooth)	No measured	[101]
Wheat	Broken wheat kernels Wheat flour Wheat bran	Kernel bread (KB) †	Wheat flour-wheat bran bread (CB)	50 g	Healthy normal-weight subjects (n = 10)	iAUC_(0–2 h)_: ↔ between test meals	iAUC GLP-1 (0–2 h, 0–6 h): ↑ CB (* vs. KB);	Hunger/appetite: ↔ between test meals	[8]
Whole-wheat	Finely milled (native and gelatinized) † Coarse (native and gelatinized) †	Whole-wheat yoghurt porridge	No external control	50 g	Subjects with T2D (n = 18)	iAUC_180 min_ ↑ Gelatinized finely milled (* vs. gelatinized, native coarse); ↑ Native finely milled (* vs. native, gelatinized coarse)	Not measured	Not measured	[100]
Wheat Maize Oats	Wheat (Whole grains, Cracked grains, Coarsely Milled flour, Finely milled flour) Maize (Whole kernels, Cracked grains, Fine flour) Oats (whole, rolled oats, and fine)	Whole/cracked/coarse/fine cereal meals †	No external control	50 g	Healthy subjects (n = 10)	AUC_180 min_: ↔ Between test meals	AUC_180 min_ Insulin: ↑ Wheat with decreasing particle size (fine vs. coarse flour +38%); ↑ Maize flour vs. cracked (+60%) and whole (+89%); Oats NS particle-size effect	Not measured	[81]
Wheat	Whole wheat kernel flour Whole meal wheat flour White wheat flour	Whole-kernel wheat bread † Wholemeal wheat bread † White wheat bread + vinegar ‡	White wheat bread	50 g	Healthy, normal-weight, overweight subjects (n = 13)	AUC_120 min_: ↔ for all the test meals	Gastric emptying: ↔ for all test meals	Satiety: ↑ with whole-kernel vs. all other test meals	[38]
Wheat Rye	Whole wheat kernels Parboiled wheat cracked wheat (bulgur) White wheat milled flour Whole wheat milled flour Rye kernels Whole rye milled flour	Wholemeal wheat bread ‡ Wholemeal rye bread ‡ Pumpernickel bread ‡ Bulgur ‡ Wheat kernels ‡ Rye kernels ‡	White wheat bread	50 g	Subjects with T2D (n = 9–12 non-insulin-dependent, n = 5–6 insulin-dependent)	GI: ↑ White wheat, wholemeal wheat bread (* vs. pumpernickel bread, bulgur, wheat, rye kernels); ↑ Pumpernickel bread (* vs. bulgur, wheat, rye kernels); ↑ Bulgur, wheat kernels (* vs. rye kernels)	Not measured	Satiety scores: ↑ at 30 min for bulgur and wheat kernels (* vs. white bread)	[120]
Wheat Barley	Bulgur Wheat flour Whole barley Barley flour	Wholemeal bread † 50% bulgur bread †/75% Bulgur bread † Bulgur † Barley flour bread † 50% Barley bread †/75% Barley bread † Barley †	White wheat bread (WB)	50 g	Subjects with T2D (n = 16)	GI ↓ with increasing whole/cracked grain	Not measured	Satiety: ↔ between test meals	[121]
Wheat Rye	Rye kernels Rye flour Oat β-glucans concentrate	Whole-kernel rye bread ‡ β-glucan rye bread ‡ Wholemeal pasta ‡	White wheat bread	50 g	Healthy subjects (n = 20)	Glucose response: ↔ between test meals	iAUC_180 min_ Insulin: ↓ in whole-kernel rye bread and pasta (* vs. white wheat bread) GIP: ↓ in all test meals (* vs. white wheat bread) GLP-1: ↔ for all test meals	Not measured	[131]
Chickpea Wheat	Whole grain (WGB) Coarse (CLP) Finely milled (CFM)	Whole wheat bread (WGB) ‡ Chickpea finely milled (CFM) ‡ Chickpea large particle (CLP) ‡	White wheat bread (WB)	50 g	Healthy normal-weight subjects (n = 15)	iAUC_180 min_^:^ ↓ for CLP (* vs. WB, CFM) ‡	iAUC_180 min_ Insulin: ↔ GLP-1: ↑ (* vs. WB) ‡	iAUC_180 min_ Hunger: ↓ for CFM, CLP (* vs. WB) Fullness: ↑ for CLP (* vs. WB) DtE ↑ for CLP (* vs. WB) ‡	[132]
Wheat	Fine wholegrain flour Medium-particle-size flour Coarse-particle-size flour	Fine-particle-size bread (Fine) † Medium-particle bread (Medium) † Coarse-particle bread (Coarse) †	White wheat bread	50 g	Healthy, normal-weight subjects (n = 42)	GI: ↔ for all test breads	Not measured	Not measured	[124]
Wheat Barley Rye Oats	White wheat flour Barley flour Intact wheat kernels Intact rye kernels Intact oat kernels Intact barley kernels Intact scalded barley kernels	White wheat coarse bread (WCB) ‡ Rye coarse bread (RCB) ‡ Oat coarse bread (OCB) ‡ Barley wholemeal (BWB)/ Barley coarse (BCB)/ Barley scalded kernel breads (BSCB) ‡	White wheat bread (WWB)	50 g	Healthy, normal weight	iAUC_120 min_: ↑ WWB (* vs. WCB, RCB, BCB, BSCB); ↑ WCB (* vs. BSCB); ↓ RCB, BCB, BSCB (* vs. OCB, BWB)	iAUC_120 min_ Insulin: ↑ WWB (* vs. WCB, RCB, OCB, BCB, BSCB); ↑ WCB (* vs. RCB, BCB, BSCB); ↓ RCB, BCB (* vs. OCB, BWB); ↑ BWB (* vs. OCB); ↓BSCB (* vs. OCB)	Satiety score: ↑ for BCB (0–15 min) (* vs. WWB)	[125]
Oats	Oat flakes Oat flours	Oat-flake porridge † Oat-flour porridge †	No external control	Not reported (30.9 carbohydrate/portion)	Healthy normal-weight-overweight subjects (n = 8)	iAUC_180 min_: ↔ Peak glucose: ↔ with flakes (* vs. flour)	Insulin response: ↑ for the oat flour porridge (* vs. oat flakes porridge) GLP-1, GIP: ↑ in the early post-prandial period for oat flour porridge (* vs. oat flakes porridge)	Fullness, hunger, satisfaction ratings: ↔ between test meals DtE: ↑ in the oat flakes porridge	[103]
Wheat	Fine roller-milled flour (FRMF) Fine stoneground flour (FSF) Coarse stoneground flour (CSF)	FRMF bread † FSF bread † CSF bread †	No external control	50 g	Subjects with known risk factors for T2D and BMI > 28 kg/m^2^ (n = 23)	iAUC_180 min_: ↔	iAUC_150 min_: Insulin ↔	Not measured	[122]
Rice	Brown rice Milled rice	Cooked brown rice (BR) ‡ Cooked white rice (MR) ‡	White wheat bread (WWB)	50 g	Healthy subjects (n = 10), subjects with T2DM (n = 9)	↓ 19.8% iAUC for BR (* vs. MR) in healthy subjects ↓ 35.2% iAUC for BR (* vs. MR) in subjects with T2D	Not measured	Not measured	[106]
Wheat Carob	Coarse wheat flour Fine wheat flour Carob seed flour White wheat flour	White wheat bread (WB) ‡ Coarse wheat bran-enriched bread (CB) ‡ Fine wheat bran-enriched bread (FB) ‡ Fine wheat bran, carob seed flour-enriched bread (CSFB) ‡	D-glucose	50 g	Healthy subjects (n = 10)	iAUC_120 min_: ↓ Coarse bran and carob-enriched breads (* vs. white bread); ↔ Fine bran vs. white bread	iAUC_120 min_: Insulin ↔ for all test breads	Hunger, DtE: ↓ correlations with GI Fullness: ↔ for all test breads	[128]
Wheat	Whole grain flour (WG) Refine flour (RG) Reconstituted fine (RC) Cracked (WG-C) Semolina (RG-S) Reconstituted coarse(RC-C)	WG-Porridge † RG-Porridge ‡ RC-Porridge † WG-C-Porridge † RG-S-Porridge ‡ RC-C-Porridge †	No external control	50 g	Healthy, normal-weight subjects (n = 16)	iAUC_120 min_: ↑ RC (* vs. WG, RG); ↔ other test meals iAUC_240 min_: ↔ between test meals	Not measured	Satiety and fullness: ↔ between test meals	[102]
Sorghum Corn Brown rice Whole wheat	Fine sorghum flour (FS) Intermediate sorghum flour (IS) Coarse sorghum flour (CS) Corn flour (CF) Rice flour (RF) Whole wheat (WW) All-purpose flour (AP)	Muffins with FS IS CS CF RF WW	Muffin with AP	20 g	Healthy subjects (n = 8)	GI: ↑ FS Muffin (* vs. IS, R muffin); ↔ other test meals	Not measured	Not measured	[126]
Wheat	Roller-milled flour 100% (RMF_100_) Roller-milled + kibbled flour (50–50%) (RMF_50_) Roller-milled + intact + kibbled flour (40–30–30%) (RMF_30_) Stone-ground flour (100%) (SGF)	RMF_100_ bread † RMF_50_ bread † RMF_30_ bread † SGF bread †	No external control	50 g	Subjects with T2D (n = 15)	iAUC_180 min_: ↑ RMF_100_ bread (* vs. RMF_40_, SGF breads); ↓ RMF_30_ breads (* vs. RMF_50_, SGF breads)	Not measured	Not measured	[15]
Rye Wheat	Whole grain Endosperm rye flour White wheat flour Rye kernels Wheat kernels	Endosperm rye bread (ERB) ‡ Endosperm rye bread + lactic acid (ERB-lac) ‡ Whole grain rye bread (WGRB) ‡ Whole grain rye bread + lactic acid (WGRB-lac) ‡ Boiled rye kernels (RK) ‡ Boiled whole grain wheat kernels (WK) ‡	White wheat bread (WWB)	50 g	Healthy, normal-weight subjects (n = 10)	GI: ↓ All test meals except ERB (* vs. WWB)	Insulin Index: ↑ for WWB (* vs. intervention breads) Ghrelin: ↓ (120–270 min) WGRB-lac (* vs. to WK) Adiponectin: ↔for all test breads	iAUC_120–270_ Satiety: ↑ RK (* vs. WB, WGRB) Energy intake RK (* vs. WB): ↓	[129]
Wheat Soybean	Kibbled grains; Chewed (C) Unchewed (U) Homogenized (H)	C, U, H breads in the following forms: Kibbled wheat bread (PB) ‡ Kibbled soya–wheat bread (SPB) ‡	White bread (WB)	40 g	Healthy subjects with a BMI of 18–35 kg/m^2^ (n = 25)	iAUC_120 min_: ↓ PB_U_ (* vs. PB_C_) †; ↔ WB, SPB ‡	Not measured	Not measured	[77]
Lentils Peas	Whole lentils Lentil flour Whole peas Pea flour	Pasta sauce of Whole lentil ‡ Lentil flour ‡ Whole pea ‡ Pea flour ‡	Glucose beverage	50 g	Subjects with T2D (n = 11) Subjects with metabolic syndrome (n = 8)	AUC_180 min_ Participants with T2D: Glucose beverage ↑ (* vs. whole lentil, whole pea test meals) Participants with metabolic syndrome: ↑ glucose beverage (* vs. all the test meals)	Not measured	Fullness: ↑ with pulse meals in subjects with Metabolic Syndrome	[117]
Oats	Steel-cut oats (SCO) Old fashioned oats (OFO) Instant oats (IO) Honey nut cheerios (HNC)	Porridges of SCO † OFO † IO † HNC ‡	Cream of rice (CR)	23 g (SCO, OFO, IO) 27.3 (HNC)	Healthy normal-weight–overweight–obese subjects (n = 30)	iAUC_180 min_: ↑ HNC, CR (* vs. SCO)	iAUC_180 min_ Insulin: ↑ IO (* vs. SCO) †	Not measured	[104]
Wheat	Coarse wheat flour (CF) Fine wheat flour (FF)	CF bread (CFB) † FF bread (FFB) †	Glucose solution	50 g	Healthy normal-weight subjects (n = 10)	iAUC_120 min_: ↓ CFB (* vs. glucose solution); ↔ CFB vs. FFB	Not reported	iAUC_120 min_ Satiety score ↔ between test meals	[130]
Oats	Coarse oat flour (COF) Whole oat flour (WOF) Fine oat flour (FOF)	Oat porridge of COF ‡ WOF ‡ FOF ‡	Commercial oat milled flour (COMF) porridge	COF 57.5 WOF 53.0 FOF 40.3 COMF 57.5	Healthy normal–overweight subjects (n = 20)	iAUC_120 min_: ↓ COF (* vs. FOF, COMF); ↓ WOF (* vs. FOF)	iAUC_120 min_ Insulin: ↑ COMF, FOF (* vs. COF, WOF)	Average appetite: ↓ COMF (* vs. COF, WOF) ↓ FOF (* vs. COF)	[105]

† Structure-focused comparison: products were compositionally comparable and particle size and/or cellular integrity was the principal manipulated variable; ‡ Confounded comparison: structural differences co-occurred with differences in botanical source, nutrient composition, available carbohydrate dose, processing conditions or other product characteristics. The observed response cannot be attributed exclusively to food structure. Symbols refer to the interpretability of the specific comparison and do not indicate study quality or statistical significance. ^a^ Available carbohydrate values are reported as provided by the original studies. When not reported, this is indicated as “Not reported”. Abbreviations: CHO, Carbohydrates, iAUC, incremental Area Under Curve, T2D, Type 2 Diabetes, BW, Body Weight, GI, Glycemic Index, GLP-1, Glucagon Like Peptide-1, GIP, Glucose-dependent insulinotropic polypeptide, PYY, Peptide Tyrosine-Tyrosine, DtE, Desire to Eat. Abbreviations: * indicates a statistically significant difference between the treatments (*p* < 0.05). The available in vitro evidence generally supports the preservation of the cellular structure, and a larger particle size can slow starch hydrolysis. However, this relationship is less consistent in human studies, where several factors beyond starch digestion, including meal composition, gastric emptying, hormonal responses, insulin sensitivity, oral processing, and individual metabolic variability, can influence the postprandial responses. The available human studies also differ in study populations, designs, sample sizes, test products, carbohydrate doses, glucose measurement methods, outcome measures, and observation periods, which limit direct comparison between studies. Most acute studies included relatively small sample sizes. Nevertheless, this does not necessarily indicate inadequate statistical power, particularly in crossover studies where sample size calculations are based on the expected within-participant differences and primary outcome variability. The use of crossover designs in several studies is an important strength, as they reduce between-participant variability and are well suited to acute postprandial comparisons. Where reported, several studies had adequate statistical power for their primary outcomes. Importantly, the available human studies appear to show more consistent effects in individuals with T2DM, whereas findings in healthy individuals are more variable [15,97,100]. This may partly reflect the more effective glucose homeostasis in metabolically healthy individuals, which may attenuate differences in postprandial glucose responses resulting from differences in starch digestion and absorption. Individual differences in mastication behavior and salivary amylase activity may further influence the extent to which structural differences are maintained during ingestion and consequently affect starch digestion and postprandial glycemia [77]. Evidence regarding the effects of food structure on gut hormone responses remains limited and heterogeneous, and further well-controlled human studies are needed to clarify these relationships [133]. Therefore, the slower starch hydrolysis observed in vitro should not be assumed to translate directly into a lower postprandial glucose response or iAUC in humans. The clinical evidence should instead be interpreted in relation to the characteristics of the studied populations and the methodological differences between studies, and whether structural changes in food and their effects on starch digestion translate into more favorable postprandial metabolic responses.

## Data Availability

No new data were created or analyzed in this study. Data sharing is not applicable to this article.

## Abbreviations

The following abbreviations are used in this manuscript:

GI	Glycemic Index
GIP	Glucose-Dependent Insulinotropic Polypeptide
GLP-1	Glucagon-Like Peptide-1
iAUC	Incremental Area Under Curve
PYY	Peptide YY
RDS	Rapidly Digestible Starch
RS	Resistant Starch
RS1	Resistant Starch Type 1
RS2	Resistant Starch Type 2
SCFA	Short-Chain Fatty Acid
SDS	Slowly Digestible Starch
SP	Swelling Power
T2DM	Type 2 Diabetes Mellitus
WHC	Water-Holding Capacity

## References

[1] Aguilera J.M. (2005). Why Food Microstructure?. J. Food Eng..

[2] Grundy M.M.L., Edwards C.H., Mackie A.R., Gidley M.J., Butterworth P.J., Ellis P.R. (2016). Re-Evaluation of the Mechanisms of Dietary Fibre and Implications for Macronutrient Bioaccessibility, Digestion and Postprandial Metabolism. Br. J. Nutr..

[3] Hall K.D., Ayuketah A., Brychta R., Cai H., Cassimatis T., Chen K.Y., Chung S.T., Costa E., Courville A., Darcey V. (2019). Ultra-Processed Diets Cause Excess Calorie Intake and Weight Gain: An Inpatient Randomized Controlled Trial of Ad Libitum Food Intake. Cell Metab..

[4] Norton J.E., Gonzalez Espinosa Y., Watson R.L., Spyropoulos F., Norton I.T. (2015). Functional Food Microstructures for Macronutrient Release and Delivery. Food Funct..

[5] Bai Y., Li X., Ji H., Wang Y., Zheng D., Wang Y., Jin Z. (2021). A Review of the Design and Architecture of Starch-Based Dietary Foods. Engineering.

[6] Kong H., Yu L., Li C., Ban X., Gu Z., Liu L., Li Z. (2022). Perspectives on Evaluating Health Effects of Starch: Beyond Postprandial Glycemic Response. Carbohydr. Polym..

[7] Bajka B.H., Pinto A.M., Ahn-Jarvis J., Ryden P., Perez-Moral N., van der Schoot A., Stocchi C., Bland C., Berry S.E., Ellis P.R. (2021). The Impact of Replacing Wheat Flour with Cellular Legume Powder on Starch Bioaccessibility, Glycaemic Response and Bread Roll Quality: A Double-Blind Randomised Controlled Trial in Healthy Participants. Food Hydrocoll..

[8] Eelderink C., Noort M.W.J., Sozer N., Koehorst M., Holst J.J., Deacon C.F., Rehfeld J.F., Poutanen K., Vonk R.J., Oudhuis L. (2017). Difference in Postprandial GLP-1 Response despite Similar Glucose Kinetics after Consumption of Wheat Breads with Different Particle Size in Healthy Men. Eur. J. Nutr..

[9] Hafiz M.S., Campbell M.D., Orsi N.M., Mappa G., Orfila C., Boesch C. (2022). Impact of Food Processing on Postprandial Glycaemic and Appetite Responses in Healthy Adults: A Randomized, Controlled Trial. Food Funct..

[10] Pälchen K., Bredie W.L.P., Duijsens D., Isaac Alfie Castillo A., Hendrickx M., Van Loey A., Raben A., Grauwet T. (2022). Effect of Processing and Microstructural Properties of Chickpea-Flours on in Vitro Digestion and Appetite Sensations. Food Res. Int..

[11] Xiong W., Devkota L., Zhang B., Muir J., Dhital S. (2022). Intact Cells: “Nutritional Capsules” in Plant Foods. Compr. Rev. Food Sci. Food Saf..

[12] Korompokis K., De Brier N., Delcour J.A. (2019). Differences in Endosperm Cell Wall Integrity in Wheat (*Triticum aestivum* L.) Milling Fractions Impact on the Way Starch Responds to Gelatinization and Pasting Treatments and Its Subsequent Enzymatic in Vitro Digestibility. Food Funct..

[13] Tagliasco M., Tecuanhuey M., Reynard R., Zuliani R., Pellegrini N., Capuano E. (2022). Monitoring the Effect of Cell Wall Integrity in Modulating the Starch Digestibility of Durum Wheat during Different Steps of Bread Making. Food Chem..

[14] Åberg S., Mann J., Neumann S., Ross A.B., Reynolds A.N. (2020). Whole-Grain Processing and Glycemic Control in Type 2 Diabetes: A Randomized Crossover Trial. Diabetes Care.

[15] Reynolds A.N., Mann J., Elbalshy M., Mete E., Robinson C., Oey I., Silcock P., Downes N., Perry T., Te Morenga L. (2020). Wholegrain Particle Size Influences Postprandial Glycemia in Type 2 Diabetes: A Randomized Crossover Study Comparing Four Wholegrain Breads. Diabetes Care.

[16] Marventano S., Vetrani C., Vitale M., Godos J., Riccardi G., Grosso G. (2017). Whole Grain Intake and Glycaemic Control in Healthy Subjects: A Systematic Review and Meta-Analysis of Randomized Controlled Trials. Nutrients.

[17] Korompokis K., Delcour J.A. (2023). Components of Wheat and Their Modifications for Modulating Starch Digestion: Evidence from in Vitro and in Vivo Studies. J. Cereal Sci..

[18] Shewry P.R., Mantila U.H., Serna-Saldivar S.O. (2023). Structure and Development of Cereal Grains. ICC Handbook of 21st Century Cereal Science and Technology.

[19] Do D.T., Singh J., Oey I., Singh H. (2019). Modulating Effect of Cotyledon Cell Microstructure on in Vitro Digestion of Starch in Legumes. Food Hydrocoll..

[20] Mishra S., Monro J. (2012). Wholeness and Primary and Secondary Food Structure Effects on in Vitro Digestion Patterns Determine Nutritionally Distinct Carbohydrate Fractions in Cereal Foods. Food Chem..

[21] Rovalino-Córdova A.M., Fogliano V., Capuano E. (2018). A Closer Look to Cell Structural Barriers Affecting Starch Digestibility in Beans. Carbohydr. Polym..

[22] Capuano E., Pellegrini N. (2019). An Integrated Look at the Effect of Structure on Nutrient Bioavailability in Plant Foods. J. Sci. Food Agric..

[23] Rovalino-Córdova A.M., Fogliano V., Capuano E. (2019). The Effect of Cell Wall Encapsulation on Macronutrients Digestion: A Case Study in Kidney Beans. Food Chem..

[24] Edwards C.H., Ryden P., Mandalari G., Butterworth P.J., Ellis P.R. (2021). Structure-Function Studies of Chickpea and Durum Wheat Uncover Mechanisms by Which Cell Wall Properties Influence Starch Bioaccessibility. Nat. Food.

[25] Bhattarai R.R., Dhital S., Wu P., Chen X.D., Gidley M.J. (2017). Digestion of Isolated Legume Cells in a Stomach-Duodenum Model: Three Mechanisms Limit Starch and Protein Hydrolysis. Food Funct..

[26] Pallares Pallares A., Alvarez Miranda B., Truong N.Q.A., Kyomugasho C., Chigwedere C.M., Hendrickx M., Grauwet T. (2018). Process-Induced Cell Wall Permeability Modulates the in Vitro Starch Digestion Kinetics of Common Bean Cotyledon Cells. Food Funct..

[27] Pallares Pallares A., Rousseau S., Chigwedere C.M., Kyomugasho C., Hendrickx M., Grauwet T. (2018). Temperature-Pressure-Time Combinations for the Generation of Common Bean Microstructures with Different Starch Susceptibilities to Hydrolysis. Food Res. Int..

[28] Edwards C.H., Maillot M., Parker R., Warren F.J. (2018). A Comparison of the Kinetics of in Vitro Starch Digestion in Smooth and Wrinkled Peas by Porcine Pancreatic Alpha-Amylase. Food Chem..

[29] Edwards C.H., Warren F.J., Campbell G.M., Gaisford S., Royall P.G., Butterworth P.J., Ellis P.R. (2015). A Study of Starch Gelatinisation Behaviour in Hydrothermally-Processed Plant Food Tissues and Implications for in Vitro Digestibility. Food Funct..

[30] Li H., Gidley M.J., Dhital S. (2019). Wall Porosity in Isolated Cells from Food Plants: Implications for Nutritional Functionality. Food Chem..

[31] Liu T., Zhen X., Lei H., Li J., Wang Y., Gou D., Zhao J. (2024). Investigating the Physicochemical Characteristics and Importance of Insoluble Dietary Fiber Extracted from Legumes: An in-Depth Study on Its Biological Functions. Food Chem. X.

[32] Drewnowski A., Detzel P., Klassen-Wigger P. (2022). Perspective: Achieving Sustainable Healthy Diets Through Formulation and Processing of Foods. Curr. Dev. Nutr..

[33] Verkempinck S.H., Hendrickx M.E., Van Loey A., Grauwet T. (2023). Engineering Strategies to Modulate Nutrient Digestion Kinetics and Bioaccessibility of Plant-Based Foods. Curr. Opin. Food Sci..

[34] Zhang C., Hu J., Xu Q., Zhang H., Li R., Guan J. (2025). Mechanical Modeling and Powder Characteristics of Wheat Milling Process: A Comparative Study of Roller, Hammer and Stone Mills. J. Agric. Food Res..

[35] Capuano E., Janssen A.E.M. (2021). Food Matrix and Macronutrient Digestion. Annu. Rev. Food Sci. Technol..

[36] Mulargia L.I., Lemmens E., Chatonidi G., Torre D.L.A., Gebruers K., Wouters A.G.B., Delcour J.A., Verbeke K. (2025). Impact of the Particle Size of Milled Wheat Ingredients of Wire-Cut Cookies on Their Glycaemic Response: A Randomised Cross-over Human Intervention Study. LWT.

[37] Mishra S., Hardacre A., Monro J. (2012). Food Structure and Carbohydrate Digestibility. Carbohydrates—Comprehensive Studies on Glycobiology and Glycotechnology.

[38] Hlebowicz J., Lindstedt S., Björgell O., Höglund P., Almér L.O., Darwiche G. (2008). The Botanical Integrity of Wheat Products Influences the Gastric Distention and Satiety in Healthy Subjects. Nutr. J..

[39] Liu S., Wang W., Lu H., Shu Q., Zhang Y., Chen Q. (2022). New Perspectives on Physiological, Biochemical and Bioactive Components during Germination of Edible Seeds: A Review. Trends Food Sci. Technol..

[40] Venn B.J., Mann J.I. (2004). Cereal Grains, Legumes and Diabetes. Eur. J. Clin. Nutr..

[41] Lin S., Gao J., Jin X., Wang Y., Dong Z., Ying J., Zhou W. (2020). Whole-Wheat Flour Particle Size Influences Dough Properties, Bread Structure and in Vitro Starch Digestibility. Food Funct..

[42] Bressiani J., Oro T., Santetti G.S., Almeida J.L., Bertolin T.E., Gómez M., Gutkoski L.C. (2017). Properties of Whole Grain Wheat Flour and Performance in Bakery Products as a Function of Particle Size. J. Cereal Sci..

[43] Tsatsaragkou K., Kara T., Ritzoulis C., Mandala I., Rosell C.M. (2017). Improving Carob Flour Performance for Making Gluten-Free Breads by Particle Size Fractionation and Jet Milling. Food Bioprocess Technol..

[44] Luhovyy B.L., Hamilton A., Kathirvel P., Mustafaalsaafin H. (2017). The Effect of Navy Bean Flour Particle Size on Carbohydrate Digestion Rate Measured in Vitro. Cereal Foods World.

[45] Marchini M., Carini E., Cataldi N., Boukid F., Blandino M., Ganino T., Vittadini E., Pellegrini N. (2021). The Use of Red Lentil Flour in Bakery Products: How Do Particle Size and Substitution Level Affect Rheological Properties of Wheat Bread Dough?. LWT.

[46] Lee D.J., Cheng F., Li D., Ding K., Carlin J., Moore E., Ai Y. (2024). Important Roles of Coarse Particles in Pasting and Gelling Performance of Different Pulse Flours under High-Temperature Heating. Food Chem..

[47] Olakanmi S.J., Jayas D.S., Paliwal J., Aluko R.E. (2024). Impact of Particle Size on the Physicochemical, Functional, and In Vitro Digestibility Properties of Fava Bean Flour and Bread. Foods.

[48] Boukid F., Vittadini E., Lusuardi F., Ganino T., Carini E., Morreale F., Pellegrini N. (2019). Does Cell Wall Integrity in Legumes Flours Modulate Physiochemical Quality and in Vitro Starch Hydrolysis of Gluten-Free Bread?. J. Funct. Foods.

[49] Cheng F., Ding K., Yin H., Tulbek M., Chigwedere C.M., Ai Y. (2023). Milling and Differential Sieving to Diversify Flour Functionality: A Comparison between Pulses and Cereals. Food Res. Int..

[50] Edwards C.H., Ryden P., Pinto A.M., van der Schoot A., Stocchi C., Perez-Moral N., Butterworth P.J., Bajka B., Berry S.E., Hill S.E. (2020). Chemical, Physical and Glycaemic Characterisation of PulseON^®^: A Novel Legume Cell-Powder Ingredient for Use in the Design of Functional Foods. J. Funct. Foods.

[51] Ahmed J., Taher A., Mulla M.Z., Al-Hazza A., Luciano G. (2016). Effect of Sieve Particle Size on Functional, Thermal, Rheological and Pasting Properties of Indian and Turkish Lentil Flour. J. Food Eng..

[52] Day L., Golding M. (2016). Food Structure, Rheology, and Texture. Encyclopedia of Food Chemistry.

[53] Drakos A., Kyriakakis G., Evageliou V., Protonotariou S., Mandala I., Ritzoulis C. (2017). Influence of Jet Milling and Particle Size on the Composition, Physicochemical and Mechanical Properties of Barley and Rye Flours. Food Chem..

[54] Berg T., Singh J., Hardacre A., Boland M.J. (2012). The Role of Cotyledon Cell Structure during in Vitro Digestion of Starch in Navy Beans. Carbohydr. Polym..

[55] Singh J., Dartois A., Kaur L. (2010). Starch Digestibility in Food Matrix: A Review. Trends Food Sci. Technol..

[56] Wursch P., Del Vedovo S., Koellreutter B. (1986). Cell Structure and Starch Nature as Key Determinants of the Digestion Rate of Starch in Legume. Am. J. Clin. Nutr..

[57] Bala V., Rajagopal S., Kumar D.P., Nalli A.D., Mahavadi S., Sanyal A.J., Grider J.R., Murthy K.S. (2014). Release of GLP-1 and PYY in Response to the Activation of G Protein-Coupled Bile Acid Receptor TGR5 Is Mediated by Epac/PLC-ε Pathway and Modulated by Endogenous H_2_S. Front. Physiol..

[58] Farooq A.M., Li C., Chen S., Fu X., Zhang B., Huang Q. (2018). Particle Size Affects Structural and in Vitro Digestion Properties of Cooked Rice Flours. Int. J. Biol. Macromol..

[59] Roman L., Gomez M., Li C., Hamaker B.R., Martinez M.M. (2017). Biophysical Features of Cereal Endosperm That Decrease Starch Digestibility. Carbohydr. Polym..

[60] Carpita N.C., Gibeaut D.M. (1993). Structural Models of Primary Cell Walls in Flowering Plants: Consistency of Molecular Structure with the Physical Properties of the Walls during Growth. Plant J..

[61] Edwards C.H., Warren F.J., Milligan P.J., Butterworth P.J., Ellis P.R. (2014). A Novel Method for Classifying Starch Digestion by Modelling the Amylolysis of Plant Foods Using First-Order Enzyme Kinetic Principles. Food Funct..

[62] Qin W., Lin Z., Wang A., Chen Z., He Y., Wang L., Liu L., Wang F., Tong L.T. (2021). Influence of Particle Size on the Properties of Rice Flour and Quality of Gluten-Free Rice Bread. LWT.

[63] Dhital S., Bhattarai R.R., Gorham J., Gidley M.J. (2016). Intactness of Cell Wall Structure Controls the in Vitro Digestion of Starch in Legumes. Food Funct..

[64] Noordraven L.E.C., Bernaerts T., Mommens L., Hendrickx M.E., Van Loey A.M. (2021). Impact of Cell Intactness and Starch State on the Thickening Potential of Chickpea Flours in Water-Flour Systems. LWT.

[65] Korompokis K., Deleu L.J., Delcour J.A. (2021). The Impact of Incorporating Coarse Wheat Farina Containing Intact Endosperm Cells in a Bread Recipe on Bread Characteristics and Starch Digestibility. J. Cereal Sci..

[66] Guo P., Yu J., Wang S., Wang S., Copeland L. (2018). Effects of Particle Size and Water Content during Cooking on the Physicochemical Properties and in Vitro Starch Digestibility of Milled Durum Wheat Grains. Food Hydrocoll..

[67] Burton P., Lightowler H.J. (2006). Influence of Bread Volume on Glycaemic Response and Satiety. Br. J. Nutr..

[68] Monro J.A., Mishra S.S., Hardacre A. (2011). Glycaemic Impact Regulation Based on Progressive Geometric Changes in Solid Starch-Based Food Particles During Digestion. Food Dig..

[69] Bhattarai R.R., Dhital S., Mense A., Gidley M.J., Shi Y.C. (2018). Intact Cellular Structure in Cereal Endosperm Limits Starch Digestion in Vitro. Food Hydrocoll..

[70] Chen X., Zhu L., Zhang H., Wu G., Cheng L., Zhang Y. (2025). Multiscale Structure Barrier of Whole Grains and Its Transformation during Heating as Starch Digestibility Controllers in Digestive Tract: A Review. Food Hydrocoll..

[71] Mandalari G., Merali Z., Ryden P., Chessa S., Bisignano C., Barreca D., Bellocco E., Laganà G., Faulks R.M., Waldron K.W. (2018). Durum Wheat Particle Size Affects Starch and Protein Digestion in Vitro. Eur. J. Nutr..

[72] Al-Rabadi G.J., Torley P.J., Williams B.A., Bryden W.L., Gidley M.J. (2012). Particle Size Heterogeneity in Milled Barley and Sorghum Grains: Effects on Physico-Chemical Properties and Starch Digestibility. J. Cereal Sci..

[73] Kathirvel P., Yamazaki Y., Zhu W., Luhovyy B.L. (2019). Glucose Release from Lentil Flours Digested in Vitro: The Role of Particle Size. Cereal Chem..

[74] Alshaalan R.A., Charalambides M.N., Edwards C.H., Ellis P.R., Alrabeah S.H., Frost G.S. (2024). Impact of Chickpea Hummus on Postprandial Blood Glucose, Insulin and Gut Hormones in Healthy Humans Combined with Mechanistic Studies of Food Structure, Rheology and Digestion Kinetics. Food Res. Int..

[75] Srv A., Mishra S., Hardacre A., Matia-Merino L., Goh K., Warren F., Monro J. (2019). Kernel Structure in Breads Reduces in Vitro Starch Digestion Rate and Estimated Glycaemic Potency Only at High Grain Inclusion Rates. Food Struct..

[76] Holm J., Björck I. (1992). Bioavailability of Starch in Various Wheat-Based Bread Products: Evaluation of Metabolic Responses in Healthy Subjects and Rate and Extent of in Vitro Starch Digestion. Am. J. Clin. Nutr..

[77] Srv A., Mishra S., Hardacre A., Matia-Merino L., Goh K., Warren F.J., Monro J.A. (2022). Glycaemic Potency Reduction by Coarse Grain Structure in Breads Is Largely Eliminated during Normal Ingestion. Br. J. Nutr..

[78] Monro J., Mishra S. (2022). In Vitro Digestive Analysis of Digestible and Resistant Starch Fractions, with Concurrent Glycemic Index Determination, in Whole Grain Wheat Products Minimally Processed for Reduced Glycaemic Impact. Foods.

[79] De La Hera E., Rosell C.M., Gomez M. (2014). Effect of Water Content and Flour Particle Size on Gluten-Free Bread Quality and Digestibility. Food Chem..

[80] Ge R., Qian X., Sun B., Ma S., Wang X. (2023). In Vitro Starch Digestibility of Maize Flour and Maize Extruded Noodles Based on Different Particle-Size Effects. J. Cereal Sci..

[81] Heaton K.W., Marcus S.N., Emmett P.M., Bolton C.H. (1988). Particle Size of Wheat, Maize, and Oat Test Meals: Effects on Plasma Glucose and Insulin Responses and on the Rate of Starch Digestion in Vitro. Am. J. Clin. Nutr..

[82] Delamare G.Y.F., Butterworth P.J., Ellis P.R., Hill S., Warren F.J., Edwards C.H. (2020). Incorporation of a Novel Leguminous Ingredient into Savoury Biscuits Reduces Their Starch Digestibility: Implications for Lowering the Glycaemic Index of Cereal Products. Food Chem. X.

[83] Yang L., Wang S., Zhang W., Zhang H., Guo L., Zheng S., Du C. (2022). Effect of Black Soybean Flour Particle Size on the Nutritional, Texture and Physicochemical Characteristics of Cookies. LWT.

[84] Sun W., Tribuzi G., Bornhorst G.M. (2023). Particle Size and Water Content Impact Breakdown and Starch Digestibility of Chickpea Snacks during in Vitro Gastrointestinal Digestion. Food Res. Int..

[85] Duijsens D., Alfie Castillo A.I., Verkempinck S.H.E., Pälchen K., Hendrickx M.E., Grauwet T. (2023). In Vitro Macronutrient Digestibility and Mineral Bioaccessibility of Lentil-Based Pasta: The Influence of Cellular Intactness. Food Chem..

[86] Yanni A.E., Iakovidi S., Vasilikopoulou E., Karathanos V.T. (2024). Legumes: A Vehicle for Transition to Sustainability. Nutrients.

[87] Byars J.A., Singh M., Kenar J.A., Felker F.C., Winkler-Moser J.K. (2021). Effect of Particle Size and Processing Method on Starch and Protein Digestibility of Navy Bean Flour. Cereal Chem..

[88] Zhang H., Sun S., Ai L. (2022). Physical Barrier Effects of Dietary Fibers on Lowering Starch Digestibility. Curr. Opin. Food Sci..

[89] Chen Z., Huang Q., Xia Q., Zha B., Sun J., Xu B., Shi Y.C. (2020). Intact Endosperm Cells in Buckwheat Flour Limit Starch Gelatinization and Digestibility in Vitro. Food Chem..

[90] Junejo S.A., Ding L., Fu X., Xiong W., Zhang B., Huang Q. (2021). Pea Cell Wall Integrity Controls the Starch and Protein Digestion Properties in the INFOGEST in Vitro Simulation. Int. J. Biol. Macromol..

[91] Li P., Dhital S., Fu X., Huang Q., Liu R., Zhang B., He X. (2020). Starch Digestion in Intact Pulse Cotyledon Cells Depends on the Extent of Thermal Treatment. Food Chem..

[92] Kulathunga J., Whitney K., Simsek S. (2023). Impact of Starter Culture on Biochemical Properties of Sourdough Bread Related to Composition and Macronutrient Digestibility. Food Biosci..

[93] Xu X., Bean S., Wu X., Shi Y.C. (2022). Effects of Protein Digestion on in Vitro Digestibility of Starch in Sorghum Differing in Endosperm Hardness and Flour Particle Size. Food Chem..

[94] Cheng F., Ding K., Yin H., Tulbek M., Fabek H., Chigwedere C.M., Anderson H., Ai Y. (2025). Influence of Coarse Particles on Nutritional Quality of Flour Ingredients: A Comparison between Cereals and Pulses. J. Food Compos. Anal..

[95] Duijsens D., Pälchen K., De Coster A., Verkempinck S.H.E., Hendrickx M.E., Grauwet T. (2022). Effect of Manufacturing Conditions on in Vitro Starch and Protein Digestibility of (Cellular) Lentil-Based Ingredients. Food Res. Int..

[96] Tinus T., Damour M., Van Riel V., Sopade P.A. (2012). Particle Size-Starch–Protein Digestibility Relationships in Cowpea (Vigna Unguiculata). J. Food Eng..

[97] Bajka B.H., Pinto A.M., Perez-Moral N., Saha S., Ryden P., Ahn-Jarvis J., van der Schoot A., Bland C., Berry S.E., Ellis P.R. (2023). Enhanced Secretion of Satiety-Promoting Gut Hormones in Healthy Humans after Consumption of White Bread Enriched with Cellular Chickpea Flour: A Randomized Crossover Study. Am. J. Clin. Nutr..

[98] Perez-Moral N., Saha S., Pinto A.M., Bajka B.H., Edwards C.H. (2023). In Vitro Protein Bioaccessibility and Human Serum Amino Acid Responses to White Bread Enriched with Intact Plant Cells. Food Chem..

[99] FAO, WHO (1998). Carbohydrates in Human Nutrition: Report of a Joint FAO/WHO Expert Consultation, Rome, 14–18 April 1997.

[100] Elbalshy M.M., Reynolds A.N., Mete E., Robinson C., Oey I., Silcock P., Haszard J.J., Perry T.L., Mann J., Te Morenga L. (2021). Gelatinisation and Milling Whole-Wheat Increases Postprandial Blood Glucose: Randomised Crossover Study of Adults with Type 2 Diabetes. Diabetologia.

[101] Edwards C.H., Grundy M.M.L., Grassby T., Vasilopoulou D., Frost G.S., Butterworth P.J., Berry S.E.E., Sanderson J., Ellis P.R. (2015). Manipulation of Starch Bioaccessibility in Wheat Endosperm to Regulate Starch Digestion, Postprandial Glycemia, Insulinemia, and Gut Hormone Responses: A Randomized Controlled Trial in Healthy Ileostomy Participants. Am. J. Clin. Nutr..

[102] Pletsch E.A., Hayes A.M.R., Chegeni M., Hamaker B.R. (2022). Matched Whole Grain Wheat and Refined Wheat Milled Products Do Not Differ in Glycemic Response or Gastric Emptying in a Randomized, Crossover Trial. Am. J. Clin. Nutr..

[103] Mackie A.R., Bajka B.H., Rigby N.M., Wilde P.J., Alves-Pereira F., Mosleth E.F., Rieder A., Kirkhus B., Salt L.J. (2017). Oatmeal Particle Size Alters Glycemic Index but Not as a Function of Gastric Emptying Rate. Am. J. Physiol. Gastrointest. Liver Physiol..

[104] Wolever T.M.S., Johnson J., Jenkins A.L., Campbell J.C., Ezatagha A., Chu Y. (2019). Impact of Oat Processing on Glycaemic and Insulinaemic Responses in Healthy Humans: A Randomised Clinical Trial. Br. J. Nutr..

[105] Zhou C.Z.C., Anderson G.H., Fan W., Vien S., Ai Y., Tulbek M., Fabek H. (2024). Increasing Particle Size of Oat Flours Decreases Postprandial Glycemia and Increases Appetite in Healthy Adults. Nutr. Res..

[106] Panlasigui L.N., Thompson L.U. (2006). Blood Glucose Lowering Effects of Brown Rice in Normal and Diabetic Subjects. Int. J. Food Sci. Nutr..

[107] Behall K.M., Scholfield D.J., Hallfrisch J. (2005). Comparison of Hormone and Glucose Responses of Overweight Women to Barley and Oats. J. Am. Coll. Nutr..

[108] Birt D.F., Boylston T., Hendrich S., Jane J.L., Hollis J., Li L., McClelland J., Moore S., Phillips G.J., Rowling M. (2013). Resistant Starch: Promise for Improving Human Health. Adv. Nutr..

[109] Noah L., Guillon F., Bouchet B., Buléon A., Molis C., Gratas M., Champ M. (1998). Digestion of Carbohydrate from White Beans (*Phaseolus vulgaris* L.) in Healthy Humans. J. Nutr..

[110] Zeng H., Safratowich B.D., Liu Z., Bussan D.D. (2026). Nutritional Composition and Functional Properties of Commonly Consumed Lentils: Impact of Commercial Lentil Types and Cooking Processes. Foods.

[111] Jenkins D.J., Jane Thorne M., Camelon K., Jenkins A., Venketeshwer Rao A., Taylor R.H., Thompson L.U., Kalmusky J., Sc B., Reichert R. (1982). Effect of Processing on Digestibility and the Blood Glucose Response: A Study of Lentils. Am. J. Clin. Nutr..

[112] O’Dea K., Nestel P.J., Antonoff L. (1980). Physical Factors Influencing Postprandial Glucose and Insulin Responses to Starch. Am. J. Clin. Nutr..

[113] Dan Ramdath D., Wolever T.M.S., Chris Siow Y., Ryland D., Hawke A., Taylor C., Zahradka P., Aliani M. (2018). Effect of Processing on Postprandial Glycemic Response and Consumer Acceptability of Lentil-Containing Food Items. Foods.

[114] Petropoulou K., Salt L.J., Edwards C.H., Warren F.J., Garcia-Perez I., Chambers E.S., Alshaalan R., Khatib M., Perez-Moral N., Cross K.L. (2020). A Natural Mutation in *Pisum sativum* L. (Pea) Alters Starch Assembly and Improves Glucose Homeostasis in Humans. Nat. Food.

[115] Tovar J., Granfeldt Y., Björck I.M. (1992). Effect of Processing on Blood Glucose and Insulin Responses to Starch in Legumes. J. Agric. Food Chem..

[116] Golay A., Coulston A.M., Hollenbeck C.B., Kaiser L.L., Würsch P., Reaven G.M. (1986). Comparison of Metabolic Effects of White Beans Processed into Two Different Physical Forms. Diabetes Care.

[117] Winham D.M., Camacho-Arriola M., Glick A.A., Hall C.A., Shelley M.C. (2025). Pea and Lentil Flours Increase Postprandial Glycemic Response in Adults with Type 2 Diabetes and Metabolic Syndrome. Foods.

[118] Anderson G.H., Liu Y., Smith C.E., Liu T.T., Nunez M.F., Mollard R.C., Luhovyy B.L. (2014). The Acute Effect of Commercially Available Pulse Powders on Postprandial Glycaemic Response in Healthy Young Men. Br. J. Nutr..

[119] Cai M., Tejpal S., Tashkova M., Ryden P., Perez-Moral N., Saha S., Garcia-Perez I., Serrano Contreras J.I., Wist J., Holmes E. (2025). Upper-Gastrointestinal Tract Metabolite Profile Regulates Glycaemic and Satiety Responses to Meals with Contrasting Structure: A Pilot Study. Nat. Metab..

[120] Jenkins D.J.A., Wolever T.M.S., Jenkins A.L., Giordano C., Giudici S., Thompson L.U., Kalmusky J., Josse R.G., Wong G.S. (1986). Low Glycemic Response to Traditionally Processed Wheat and Rye Products: Bulgur and Pumpernickel Bread. Am. J. Clin. Nutr..

[121] Jenkins D.J.A., Wesson V., Wolever T.M.S., Jenkins A.L., Kalmusky J., Guidici S., Csima A., Josse R.G., Wong G.S. (1988). Wholemeal versus Wholegrain Breads: Proportion of Whole or Cracked Grain and the Glycaemic Response. BMJ Br. Med. J..

[122] Mete E., Haszard J., Perry T., Oey I., Mann J., Te Morenga L. (2021). Effect of Wholegrain Flour Particle Size in Bread on Glycaemic and Insulinaemic Response among People with Risk Factors for Type 2 Diabetes: A Randomised Crossover Trial. Nutrients.

[123] Behall K.M., Scholfield D.J., Hallfrisch J. (1999). The Effect of Particle Size of Whole-Grain Flour on Plasma Glucose, Insulin, Glucagon and Thyroid-Stimulating Hormone in Humans. J. Am. Coll. Nutr..

[124] Lanzerstorfer P., Rechenmacher E., Lugmayr O., Stadlbauer V., Höglinger O., Andreas V., Weghuber J. (2018). Effects of Various Commercial Whole-Grain Breads on Postprandial Blood Glucose Response and Glycemic Index in Healthy Subjects. Austin J. Clin. Med..

[125] Liljeberg H., Granfeldt Y., Bjorck I. (1992). Metabolic Responses to Starch in Bread Containing Intact Kernels versus Milled Flour. Eur. J. Clin. Nutr..

[126] Navarro V., Miranda J., Churruca I., Pruett A., Aramouni F.M., Bean S.R., Haub M.D. (2023). Effect of Flour Particle Size on the Glycemic Index of Muffins Made from Whole Sorghum, Whole Corn, Brown Rice, Whole Wheat, or Refined Wheat Flours. Foods.

[127] Mere E., Ngāpuhi M. (2021). Effect of Grain Particle Size of Wholegrain Foods on the Postprandial Glycaemic Response. Ph.D. Thesis.

[128] Papakonstantinou E., Chaloulos P., Papalexi A., Mandala I. (2018). Effects of Bran Size and Carob Seed Flour of Optimized Bread Formulas on Glycemic Responses in Humans: A Randomized Clinical Trial. J. Funct. Foods.

[129] Rosén L.A.H., Östman E.M., Björck I.M.E. (2011). Effects of Cereal Breakfasts on Postprandial Glucose, Appetite Regulation and Voluntary Energy Intake at a Subsequent Standardized Lunch; Focusing on Rye Products. Nutr. J..

[130] Yaregal Z., Baye K., Solomon W.K. (2022). The Influence of Dough Kneading Time and Flour Particle Size Distribution on White Bread Structure, Glycemic Response and Aspects of Appetite. Clin. Nutr. ESPEN.

[131] Juntunen K.S., Niskanen L.K., Liukkonen K.H., Poutanen K.S., Holst J.J., Mykkänen H.M. (2002). Postprandial Glucose, Insulin, and Incretin Responses to Grain Products in Healthy Subjects. Am. J. Clin. Nutr..

[132] Kanata M.C., Yanni A.E., Koliaki C., Anastasiou I.A., Tentolouris N., Karathanos V.T. (2025). Impact of Flour Particle Size and Origin on the Bread Structure and the Postprandial Glycemic, Insulinemic and Appetite Responses in Healthy Adults. Food Funct..

[133] Cai M., Dou B., Pugh J.E., Lett A.M., Frost G.S. (2021). The Impact of Starchy Food Structure on Postprandial Glycemic Response and Appetite: A Systematic Review with Meta-Analysis of Randomized Crossover Trials. Am. J. Clin. Nutr..

[134] Järvi A.E., Björck I.E., Karlström B.E., Asp N.G.L., Granfeldt Y.E., Vessby B.O.H. (1999). Improved Glycemic Control and Lipid Profile and Normalized Fibrinolytic Activity on a Low-Glycemic Index Diet in Type 2 Diabetic Patients. Diabetes Care.

[135] Philip Karl J., Meydani M., Barnett J.B., Vanegas S.M., Goldin B., Kane A., Rasmussen H., Saltzman E., Vangay P., Knights D. (2017). Substituting Whole Grains for Refined Grains in a 6-Wk Randomized Trial Favorably Affects Energy-Balance Metrics in Healthy Men and Postmenopausal Women. Am. J. Clin. Nutr..

[136] Tosh S.M., Chu Y.F. (2015). Systematic Review of the Effect of Processing of Whole-Grain Oat Cereals on Glycaemic Response. Br. J. Nutr..

[137] Musa-Veloso K., Noori D., Venditti C., Poon T., Johnson J., Harkness L.S., O’Shea M., Chu Y. (2021). A Systematic Review and Meta-Analysis of Randomized Controlled Trials on the Effects of Oats and Oat Processing on Postprandial Blood Glucose and Insulin Responses. J. Nutr..

[138] Musa-Veloso K., Poon T., Harkness L.S., O’Shea M., Chu Y. (2018). The Effects of Whole-Grain Compared with Refined Wheat, Rice, and Rye on the Postprandial Blood Glucose Response: A Systematic Review and Meta-Analysis of Randomized Controlled Trials. Am. J. Clin. Nutr..

[139] Fardet A., Leenhardt F., Lioger D., Scalbert A., Rémésy C. (2006). Parameters Controlling the Glycaemic Response to Breads. Nutr. Res. Rev..

[140] Eelderink C., Schepers M., Preston T., Vonk R.J., Oudhuis L., Priebe M.G. (2012). Slowly and Rapidly Digestible Starchy Foods Can Elicit a Similar Glycemic Response Because of Differential Tissue Glucose Uptake in Healthy Men. Am. J. Clin. Nutr..

